# Repeated translocation of a gene cassette drives sex-chromosome turnover in strawberries

**DOI:** 10.1371/journal.pbio.2006062

**Published:** 2018-08-27

**Authors:** Jacob A. Tennessen, Na Wei, Shannon C. K. Straub, Rajanikanth Govindarajulu, Aaron Liston, Tia-Lynn Ashman

**Affiliations:** 1 Department of Integrative Biology, Oregon State University, Corvallis, Oregon, United States of America; 2 Department of Biological Sciences, University of Pittsburgh, Pittsburgh, Pennsylvania, United States of America; 3 Department of Botany and Plant Pathology, Oregon State University, Corvallis, Oregon, United States of America; Indiana University, United States of America

## Abstract

Turnovers of sex-determining systems represent important diversifying forces across eukaryotes. Shifts in sex chromosomes—but conservation of the master sex-determining genes—characterize distantly related animal lineages. Yet in plants, in which separate sexes have evolved repeatedly and sex chromosomes are typically homomorphic, we do not know whether such translocations drive sex-chromosome turnovers within closely related taxonomic groups. This phenomenon can only be demonstrated by identifying sex-associated nucleotide sequences, still largely unknown in plants. The wild North American octoploid strawberries (*Fragaria*) exhibit separate sexes (dioecy) with homomorphic, female heterogametic (ZW) inheritance, yet sex maps to three different chromosomes in different taxa. To characterize these turnovers, we identified sequences unique to females and assembled their reads into contigs. For most octoploid *Fragaria* taxa, a short (13 kb) sequence was observed in all females and never in males, implicating it as the sex-determining region (SDR). This female-specific “SDR cassette” contains both a gene with a known role in fruit and pollen production and a novel retrogene absent on Z and autosomal chromosomes. Phylogenetic comparison of SDR cassettes revealed three clades and a history of repeated translocation. Remarkably, the translocations can be ordered temporally due to the capture of adjacent sequence with each successive move. The accumulation of the “souvenir” sequence—and the resultant expansion of the hemizygous SDR over time—could have been adaptive by locking genes into linkage with sex. Terminal inverted repeats at the insertion borders suggest a means of movement. To our knowledge, this is the first plant SDR shown to be translocated, and it suggests a new mechanism (“move-lock-grow”) for expansion and diversification of incipient sex chromosomes.

## Author summary

Sex chromosomes frequently restructure themselves during organismal evolution, often becoming highly differentiated. This dynamic process is poorly understood for most taxa, especially during the early stages typical of many dioecious flowering plants. We show that in wild strawberries, a female-specific region of DNA is associated with sex and has repeatedly changed its genomic location, each time increasing the size of the hemizygous female-specific sequence on the W sex chromosome. This observation shows, for the first time to our knowledge, that plant sex regions can “jump” and suggests that this phenomenon may be adaptive by gathering and locking new genes into linkage with sex. This conserved and presumed causal sex-determining sequence, which varies in both genomic location and degree of differentiation, will facilitate future studies to understand how sex chromosomes first begin to differentiate.

## Introduction

Sex chromosomes can be a strikingly diverse and evolutionarily labile component of eukaryotic genomes [1]. The defining feature of a sex chromosome, the sex-determining region (SDR), has experienced similar restructuring in multiple independent instances of autosomes evolving into heteromorphic sex chromosomes [2]. Specifically, recombination is suppressed, and an increasingly greater proportion of the chromosome becomes hemizygous, which is thought to involve existing and/or newly acquired linkage to loci under sexually antagonistic selection [3]. The mechanisms of this chromosome restructuring may involve modifying crossover sites and/or successive inversions of the SDR or translocations of large or small sequences on and off the sex chromosome [3,4]. Turnovers that change the genomic location of the SDR have been revealed in the evolution of animal sex-determining systems [1,5–8], where they may be important drivers of sexual dimorphism and speciation [9,10]. While theory on the processes driving these transitions is growing [11–14], few systems exist in which the mechanisms of turnovers can be empirically inferred [15–17].

Fundamental questions about SDR turnovers therefore remain unanswered. Do turnovers typically involve mutations in new loci that take control of an existing sex-determining mechanism [18,19], functionally independent mutations [20], or translocations of the existing sex-determining gene(s) to new chromosomes [21–24]? Similarly, do turnovers typically restart the process of SDR divergence, maintaining “ever-young” sex chromosomes [25], or do they contribute to increasing chromosome heteromorphy via loss or gain of sequence [11,14,26]? And ultimately, is there an adaptive basis for these turnovers? Although master sex-determining genes like *SRY* and *DMRT1* are highly conserved in some animal systems, the causal SDR loci or gene cassettes remain unknown for most dioecious eukaryotes [27]. Even less is known about the temporal order of turnovers in any taxon and thus directional trends in sex-chromosomal rearrangement [2].

Turnovers of SDRs are likely to be quite common in plants, in which genetic control of sex appears to be poorly conserved [28,29]. Flowering plant SDRs may be diverse because dioecy (separate males and females) has evolved repeatedly from hermaphroditism (combined male and female function) and many sex chromosomes are relatively young and homomorphic [28,29,30]. Additionally, approximately one-third of flowering plant species are estimated to have a recent polyploid ancestry [31]. These whole-genome duplications provide a larger substrate for potential sex-determining genes or rearrangements [32]. Yet despite the potential of dioecious plants for yielding evolutionary insights, there are few systems with mapped SDRs [28,29] or known causal genes [33,34], although long-standing theory predicts that two linked genes, one controlling male function and one controlling female function, are involved [1,35]. Moreover, even when observed, the pattern and mechanism of turnovers remain entirely unexplored.

**Fig 1:**
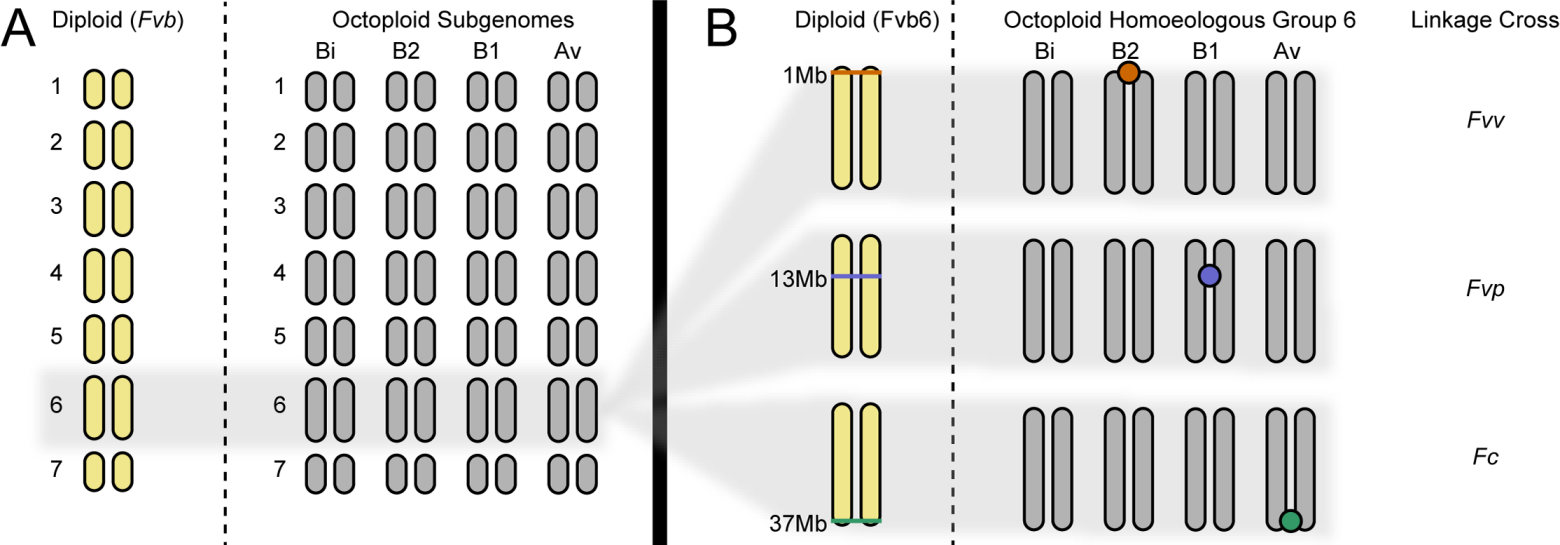
Polyploid composition and map locations of the SDR in octoploid *Fragaria*. (A) There are seven haploid *Fragaria* chromosomes. Diploids (e.g., *F*. *vesca* ssp. *bracteata*, used to generate the reference genome *Fvb*) have two copies of each. Octoploids have eight copies of each, within four homoeologous subgenomes (Bi, B2, B1, Av) showing high synteny with each other and with *Fvb*. (B) In multiple independent linkage crosses across octoploid taxa, the SDR (colored circles) had been previously mapped to three locations on different chromosomes of homoeologous group 6, corresponding to three positions (1 Mb, 13 Mb, or 37 Mb) on Fvb6. The “Linkage Cross” column indicates the taxon in which each SDR has been fine-mapped. Sex always showed ZW inheritance, but no sex-specific sequence had been previously identified. *Fc*, *F*. *chiloensis*; *Fvb*, diploid reference genome assembly informed by *F*. *vesca* ssp. *bracteata*; *Fvp*, *F*. *virginiana* ssp. *platypetala*; *Fvv*, *F*. *virginiana* ssp. *virginiana*; Mb, megabase; SDR, sex-determining region.

The octoploid (8*x*) strawberries (*Fragaria*) stand out as model system for studying plant sex chromosomes [36–40] and polyploidy [36,41] in an evolutionary context because they show recently evolved dioecy from within a group of closely related, predominantly hermaphroditic diploid (2*x*) taxa. The octoploid taxa all possess homomorphic, female heterogametic (ZW) sex chromosomes with a single SDR explaining the majority of variation in male and female function, though the degree of sexual dimorphism varies across taxa [36,39,42–45]. Male function (sterile versus fertile), in particular, is a binary trait showing simple Mendelian inheritance (1:1). Male sterility (“female”) is dominant to male fertility (“male”), determined entirely by the SDR, and here we use it to define sex phenotype. All octoploid species share a recent polyploid origin involving four diploid ancestors (now coexisting as “subgenomes” [Av, Bi, B1, and B2] within the octoploid genome, Fig 1*A*) [41,46]. The homologous chromosomes from each subgenome (homoeologs) are genetically distinct and are inherited disomically. Nevertheless, homoeologs show high synteny with each other and with the *Fragaria* reference genome (“*Fvb*”) derived from the hermaphroditic diploid *F*. *vesca* with seven haploid chromosomes (named Fvb1 through Fvb7, Fig 1*A*) [41]. Therefore, the octoploids have seven homoeologous groups, each with eight chromosomes (2*N* = 8*x* = 56). The approximately 700 megabase (Mb) octoploid genome is slightly smaller, however, than four times the approximately 200 Mb diploid *Fvb* reference genome, likely due to numerous small deletions [41,47]. The diploid and octoploid genomes are largely collinear [41], and we refer to all genome positions by their location along *Fvb* chromosomes in Mb.

The SDR of *Fragaria* octoploids has been mapped in three geographically distinct octoploid taxa (here in order from eastern to western North America): *F*. *virginiana* ssp. *virginiana* [42], *F*. *virginiana* ssp. *platypetala* [40], and *F*. *chiloensis* [39,43] (Tables 1 and 2*A*). Each SDR occurs at a unique section of a chromosome from the same homoeologous group, i.e., the group that corresponds to Fvb6 in the diploid reference, but each from a different subgenome (Fig 1*B*) [40]. Specifically, the mapped SDR locations match Fvb6 position 1 Mb on subgenome B2 in a cross of two *F*. *virginiana* ssp. *virginiana* parents ([42], results herein), 13 Mb on subgenome B1 in a cross of two *F*. *virginiana* ssp. *platypetala* parents [40], and 37 Mb on subgenome Av in three crosses involving pairs of *F*. *chiloensis* parents [39,43] (Table 2*A*). Moreover, genetic maps in the natural hybrid *(F*. *× ananassa* ssp. *cuneifolia*) of two of these taxa corroborate these map locations [48] (Table 2*A*). Though the chromosomes harboring the various SDRs are all homoeologous, they are distinct: *Fragaria* subgenomes show little evidence of recombination with each other [41], and the positions of the various SDR locations are too far apart (several Mb) for normal recombination (Fig 1*B*). All SDRs occur far from centromeres in gene-dense regions, and although early stages of recombination suppression may be evolving, pseudoautosomal recombination still occurs between the Z and W along most of their lengths, allowing for fine-scale mapping [39]. The recent evolutionary origin of dioecy and the extensive recombination still occurring on the sex chromosomes suggest that there is very little sex-specific sequence other than the causal gene(s). However, despite extensive previous work mapping the chromosomal locations of *Fragaria* SDRs [39,40,42,43,48–50] as well as conjecture that autosome Fvb6 may possess sexually antagonistic genes that predispose it to become a sex chromosome [51]—as seen in other systems [52–54]—no candidate causal genes have been identified, and nothing is known of the molecular mechanism beyond very broad inferences (e.g., that control is nuclear rather than cytoplasmic). Therefore, identifying sex-determining gene(s) and inferring whether they are shared across the octoploid *Fragaria* will provide a unique opportunity for testing whether sex chromosome turnovers represent translocations of the same SDR.

**Table 1:** *Fragaria* taxa sequenced and SDR positions mapped in linkage crosses.

Species or Subspecies	Female Individuals^a^	Linkage Cross Mothers^b^	Male Individuals^c^	SDR (Mb, Subgenome)^d^
*F*. *virginiana* ssp. *virginiana*	9	1	8	Fvb6 (1, B2)
*F*. *virginiana* ssp. *platypetala*	9	1	8	Fvb6 (13, B1)
*F*. *chiloensis*^e^	9	3	11	Fvb6 (37, Av)
*F*. *virginiana* ssp. *glauca*	3	0	0	N/A
*F*. × *ananassa* ssp. *cuneifolia*	1	1	2	Fvb6 (13, B1)

^a^Number of female plants with whole genome sequenced.

^b^Of the female plants with whole genome sequenced, the number of mothers from previous linkage crosses in which the SDR has been mapped.

^c^Number of male (male-fertile) plants with whole genome sequenced.

^d^Mapped in previous studies [39,40,42,43,48] or in this study (Table 2).

^e^*F*. *chiloensis* ssp. *pacifica*.

**Abbreviations:** *Fvb*, diploid reference genome assembly informed by *F*. *vesca* ssp. *bracteata*; Mb, megabase; N/A, not applicable; SDR, sex-determining region.

**Table 2:** Elucidating the three SDR locations in octoploid *Fragaria*.

(A) Prior Results (Genetic Mapping and Subgenome Identification)				(B) New Results (Genetic Mapping and Whole-Genome Sequencing)				
Map Location	Subgenome	Taxa Mapped in Linkage Crosses	Reference	Refined Map Location	Clade	Taxa Observed (% of Females per Taxon in Clade)	SDR Sequences	Hemizygous Portion
Fvb6: 0–5.5 Mb	B2	*F*. *virginiana* ssp. *virginiana*	[42]	Fvb6: 1.630–1.770 Mb	α	*F*. *virginiana* ssp. *virginiana* (78%); *F*. *virginiana* ssp. *platypetala* (33%); *F*. *virginiana* ssp. *glauca* (33%)	SDR cassette	*RPP0W* (1 kb)
Fvb6: 12.935–13.230 Mb	B1	*F*. *virginiana* ssp. *platypetala*; *F*. *× ananassa* ssp. *cuneifolia*	[40,48]	N/A	β	*F*. *virginiana* ssp. *platypetala* (22%); *F*. *× ananassa* ssp. *cuneifolia* (100%)	SDR cassette; flanking	SDR cassette (13 kb)
Fvb6: 37.428–37.708 Mb	Av	*F*. *chiloensis*; *F*. *× ananassa* ssp. *cuneifolia*	[39,43,48]	N/A	γ	*F*. *virginiana* ssp. *virginiana* (22%); *F*. *virginiana* ssp. *platypetala* (44%); *F*. *chiloensis* (100%)	SDR cassette; flanking; outer	SDR cassette plus flanking (23 kb)

**Abbreviations:** *Fvb*, diploid reference genome assembly informed by *F*. *vesca* ssp. *bracteata*; Mb, megabase; N/A, not applicable; SDR, sex-determining region.

Here, we use whole-genome sequencing and molecular evolutionary analysis of multiple octoploid *Fragaria* taxa to characterize and compare SDRs that are found on different chromosomes (Fig 1*B* and Table 2*B*). Our goal is to determine whether a single W-specific sequence has translocated among genomic locations. We find an “SDR cassette” shared by females across taxa and never detected in male plants. The SDR cassette contains two putatively functional sex-determining genes and has moved at least twice, together with flanking sequences that reveal the order of the translocation events. Because the moved regions are hemizygous, each translocation has created a wider hemizygous region than formerly existed. Therefore, we report the first case, to our knowledge, of a repeatedly translocating SDR in plants and propose a new hypothesis for sex-chromosome differentiation.

## Results and discussion

### A female-specific SDR cassette shared across taxa

To identify sequence unique to the W chromosome(s), we sequenced the complete genomes of 31 female and 29 male plants in five octoploid taxa (Tables 1 and S1 and S1 Fig; range of coverage relative to the haploid reference genome = 16–57×; median = 33×). These represent the North American range of the octoploid *Fragaria* and include the parents of the crosses used to map sex determination (Table 2A and S2 Fig) [36,39,40,43,48]. From these reads, we then identified sequence (“31-mers”: 31 bp motifs, the longest computationally feasible size under our particular pipeline, S1 Fig) seen in females but never in males (S2 Table). Fewer than 5% of these female-specific 31-mers aligned to more than one location in the *F*. *vesca* reference genome (*Fvb*), suggesting that the female-specific sequence is not highly repetitive. In 29 out of 31 females, we observe similar female-specific sequence. The exceptions here (2 out of 31 females) are both *F*. *virginiana* ssp. *glauca* plants, which originated from a distinct geographic region from all other samples (i.e., the Rocky Mountains, S1 Table and S2 Fig) and could carry distinct versions of this sequence or possibly possess nonhomologous SDR(s). We did not observe all shared female-specific sequence in the remaining 29 females, as expected owing to missing data due to our low sequencing coverage (2–7× per octoploid chromosome). Still, these 29 females all possess female-specific 31-mers aligning to the same 2 kb window on Fvb7 position 18 Mb (S3 Fig). They also possess sequence overlapping a single site homologous to Fvb6 position 1 Mb, where these octoploid females possess a 23 bp “diagnostic deletion” not seen in the diploid hermaphrodite *F*. *vesca* (*Fvb*) or any of the 29 male plants (S3 Fig). In contrast to the female-specific sequence found, male-specific 31-mers were rarely seen (S2 Table), as expected because Z chromosomes are present in both males and females, suggesting that our method yields a very small number of false-positive 31-mers. Moreover, while female-specific sequence is shared across the octoploid taxa, the SDRs of these plants maps to three different genomic locations. This suggests that translocations are likely involved, which demands further characterization of female-specific sequence for confirmation (see below).

**Fig 2:**
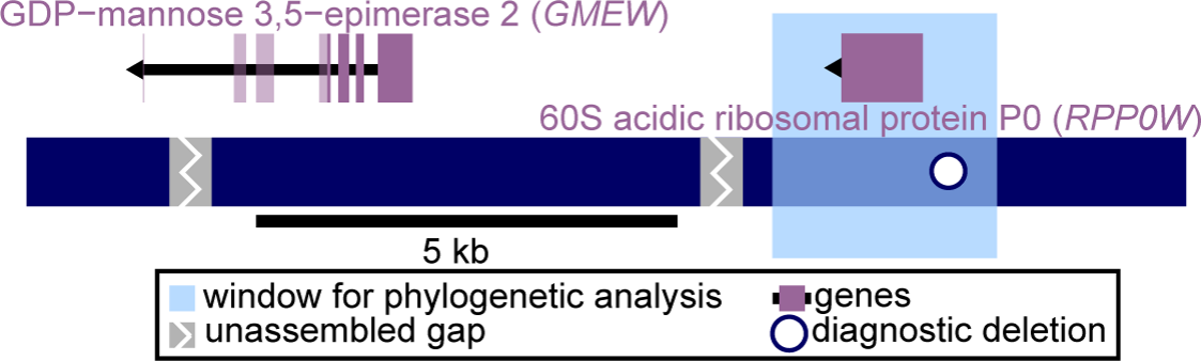
The SDR cassette. The “SDR cassette,” a 13 kb haplotype occurring in nearly all females (29/31) and never in males (0/29), was assembled from reads containing shared female-specific sequence (S2 Table). This cassette contains two predicted genes, *GMEW* and *RPP0W* (direction of transcription indicated by arrowheads); *GMEW* exons downstream of the variable stop codon are faded. The locations of two assembly gaps and the diagnostic deletion are also indicated. The light blue rectangle indicates the 2.7 kb window used in phylogenetic analysis (Fig 3). SDR, sex-determining region.

To assess and annotate the SDR, we assembled the shared female-specific sequence, generating three contigs totaling 13 kb in length. These contigs were ordered and oriented into a unified W-specific haplotype, the SDR cassette (Fig 2), by using highly similar autosomal and Z chromosome sequences as scaffolds. Specifically, most (10.4 kb) of the SDR cassette could be aligned (98% similarity) to Z chromosome bacterial artificial chromosomes (BACs) obtained from *F*. *virginiana* ssp. *virginiana*, originating from the maternal linkage cross parent at the fine-mapped SDR location from that cross (S4 and S5 Figs). Most of this sequence (8.6 kb) could also be aligned (93% similarity) to the diploid (*F*. *vesca*) reference genome at the fine-mapped location of Fvb6 position 1 Mb. A 1.2 kb segment of the SDR cassette was not homologous to Fvb6 but instead showed 99% similarity to Fvb7 position 18 Mb. Therefore, the W-specific SDR cassette is relatively short and shows homology to multiple sections of the genome.

Only two coding genes—annotated as GDP-mannose 3,5-epimerase 2 (here *GMEW*) and 60S acidic ribosomal protein P0 (here *RPP0W*)—were identified in the SDR cassette (Fig 2). *GMEW* homologs occur on the Z chromosome BACs (99% similarity) and at Fvb6 position 1 Mb (98% similarity). GDP-mannose 3,5-epimerase converts GDP-mannose to GDP-L-galactose in vitamin C and cell wall biosynthesis [55,56], affecting fruit development in *Fragaria* [57,58] and pollen production in other plants [56]. In some females, *GMEW* has a premature stop codon shortening the coding sequence from 376 to 222 residues. Whereas *GMEW* is a plausible sex-determining candidate, the stop codon polymorphism may suggest a variable role among females. In contrast, the second gene, *RPP0W*, falls within a 1.2 kb W-specific insertion that shows 99% similarity to a gene at Fvb7 position 18 Mb and is thus responsible for the female-specific 31-mers homologous to that location (S3 Fig). However, it lacks that gene’s four introns, suggesting that it is a cDNA resulting from retrotransposition. *RPP0W* sequences across the *Fragaria* taxa studied here form a monophyletic group with respect to this autosomal paralog and other autosomal paralogs (S6 Fig and S1 Data), a finding that is consistent with a single SDR origin. Ribosomal proteins are essential for polypeptide synthesis and are often retrotransposed [59]. In plants they can affect processes from development to stress response [60], with mutations sometimes acting dominantly [61], as expected for the first mutation in a female heterogamic (ZW) system [1]. In rice, the overaccumulation of ubiquitin fusion ribosomal protein L40 results in defective pollen and male sterility [62]. In diploid hermaphroditic *F*. *vesca*, both *RPP0W* and *GMEW* homologs show decreasing expression during anther development and even lower expression within pollen [63], but expression profiles in octoploids remain to be characterized. Neither gene family (of *GMEW* nor *RPP0W*) has been directly implicated in sex determination, but many pathways could potentially affect plant sex functions [64,65].

In classic two-gene SDR models, one gene affects male function and another female function [35]. Previous quantitative trait locus (QTL) mapping has shown that the *Fragaria* SDR affects both male and female function [36,39,40] and shows differential recombination rates in ZZ versus ZW individuals [39]. However, we cannot yet conclude that there are two functional, non-recombining genes because a single master regulator could also perform both roles [33] and additional modifiers of female function could have evolved. Moreover, in addition to the two genes, there is the diagnostic deletion and two repetitive unassembled gaps within the SDR (Fig 2), which, though apparently noncoding, could also be functional motifs. Regardless, what is striking here is that an SDR cassette (W-specific) is shared across females from different taxa and populations where it occurs at multiple genomic locations (Fig 1*B*).

### Inferring the translocation history

**Fig 3:**
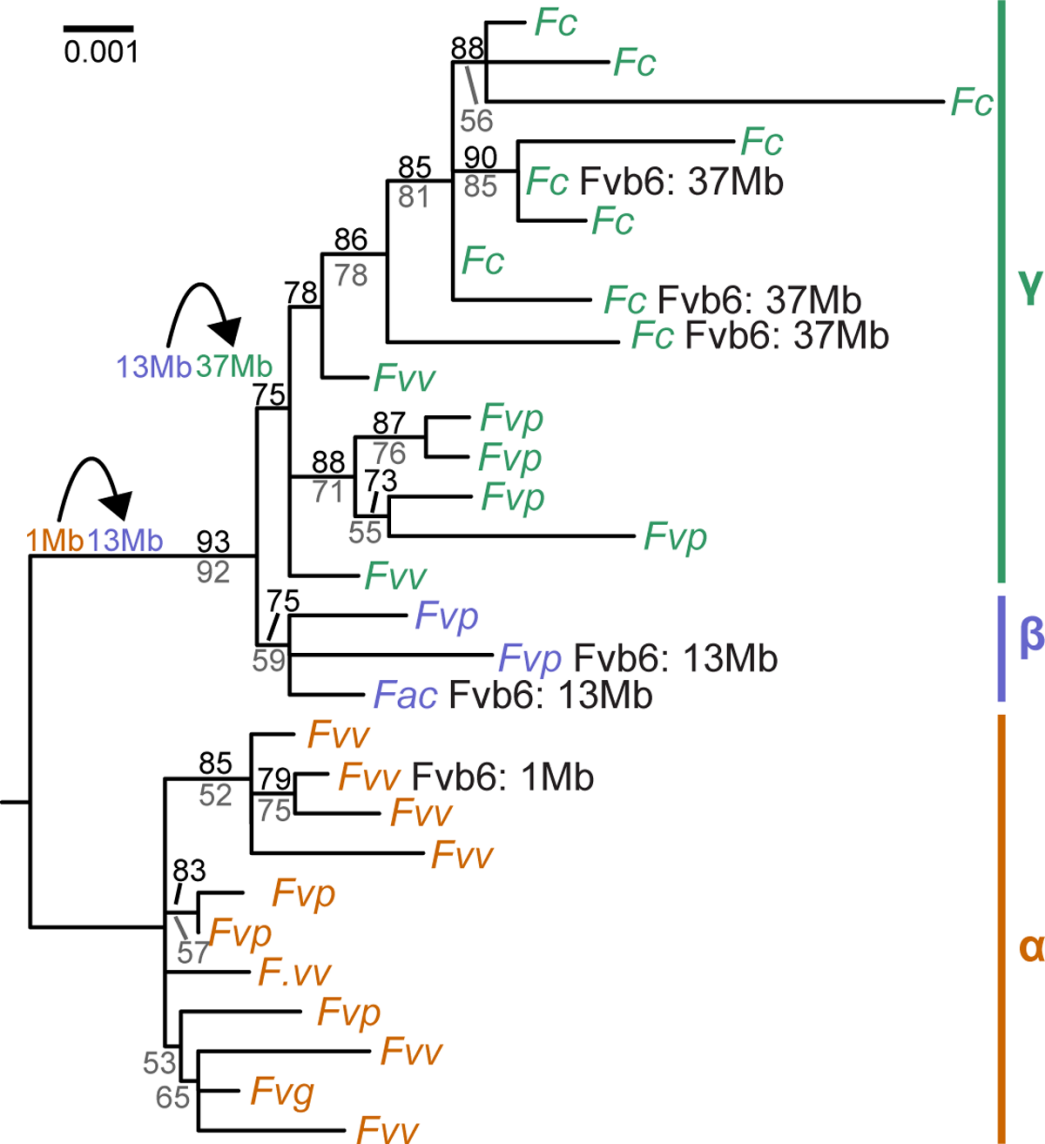
Phylogenetic history of the hemizygous female-specific SDR cassette. Phylogeny of the central 2.7 kb of the hemizygous SDR cassette (blue box, Fig 2; 0%–30% missing data; mean = 16%) from females of several octoploid strawberries (S1 Table). Three major clades, α, β, and γ, are revealed. Shimodaira-Hasegawa-like (black, above branches) and bootstrap (grey, below branches) support is shown if >50%. SDRs that have been mapped are noted along with the location of the SDR. A pseudo-outgroup sequence (not shown) was generated with consistent homology to the W haplotype along this 2.7 kb portion, by concatenation of sequence from *F*. *vesca* reference genome Fvb6 position 1 Mb and orthologous Z chromosome BAC sequence, together with 1.2 kb of sequence from Fvb7 position 18 Mb corresponding to the closest autosomal paralog of *RPP0W*. The close evolutionary relationship between the β and γ clades is consistent with the inferred history of translocations, indicated at two points with black curved arrows to the left of the phylogeny. BAC, bacterial artificial chromosome; *Fac*, *F*. *× ananassa* ssp. *cuneifolia*; *Fc*, *F*. *chiloensis*; *Fvb*, diploid reference genome assembly informed by *F*. *vesca* ssp. *bracteata*; *Fvg*, *F*. *virginiana* ssp. *glauca*; *Fvp*, *F*. *virginiana* ssp. *platypetala*; *Fvv*, *F*. *virginiana* ssp. *virginiana*; Mb, megabase; SDR, sex-determining region.

To infer the evolutionary history of the shared SDR cassette, we reconstructed the phylogeny of a 2.7 kb portion overlapping *RPP0W* and the diagnostic deletion (Fig 2) in the 29 females with the SDR cassette (Fig 3 and S2 Data). These W-specific sequences resolved into three distinct and well-supported (≥75% Shimodaira-Hasegawa–like support) clades: α, β, and γ (Fig 3 and Table 2). Notably, each of the three SDR map locations (Fig 1*B*) is associated with a single clade (Fig 3). The SDRs from the *F*. *virginiana* ssp. *virginiana* female for which male sterility was fine-mapped to Fvb6 position 1 Mb (S4 Fig and Table 2*B*)—and those of most other *F*. *virginiana* ssp. *virginiana* females—were in the α clade. SDRs of the β clade include two from females for which male sterility has been previously mapped to Fvb6 position 13 Mb (Table 2*B*) [40,48]. The SDRs that form the γ clade included those from all three *F*. *chiloensis* females for which male sterility maps to Fvb6 position 37 Mb (Table 2*B*) [39,43] and the remaining six *F*. *chiloensis* females as well as a few from females of *F*. *virginiana* ssp. *virginiana* and *F*. *virginiana* ssp. *platypetala*. The overall topology, with *F*. *chiloensis* nested within *F*. *virginiana*, reflects the inferred evolutionary history of these taxa, which are not reciprocally monophyletic [46]. The β and γ clades are sister to each other with strong support (93% Shimodaira-Hasegawa–like support; 92% bootstrap support; Fig 3), suggesting that these SDRs (Fvb6 positions 13 Mb and 37 Mb) may be more closely related, whereas those in the α clade (Fvb6 position 1 Mb) are more distantly related and represent the source of the homologous sequence shared across all clades (S3 Fig).

**Fig 4:**
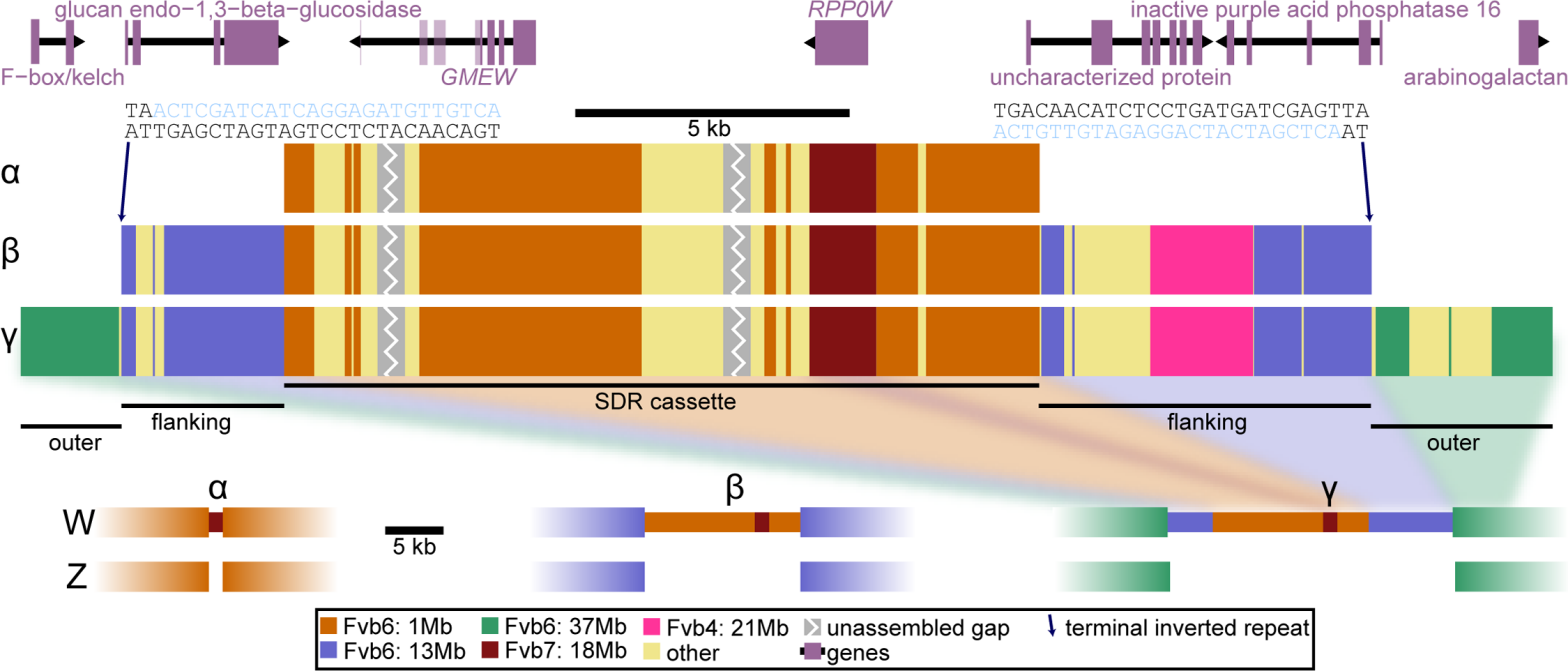
W-specific SDR haplotype composition across octoploid *Fragaria*. Top: there are seven predicted genes in the longest haplotype (S3 Table), including two shared by all females (*GMEW* and *RPP0W*, Fig 2). Middle: all three clades (α, β, and γ) share the SDR cassette, suggesting that it is the oldest and that Fvb6 position 1 Mb is the original SDR position. Clades β and γ also share the flanking sections, suggesting a translocation to Fvb6 position 13 Mb. Only clade γ possesses the outer section, consistent with a second translocation to Fvb6 position 37 Mb unique to this clade. At either ends of the flanking sequences, terminal inverted repeats (blue nucleotides) are adjacent to target-site duplications (TA dinucleotide), a pattern consistent with transposon-mediated movement of this section. Bottom: inferred size and composition of the hemizygous W-specific insertion in each of the three clades, α, β, and γ. Z chromosome composition is inferred from the *Fvb* reference genome and Z-specific sequence obtained from BACs of a maternal *F*. *virginiana* ssp. *virginiana* linkage cross parent in clade α (S4 Fig). BAC, bacterial artificial chromosome; *Fvb*, diploid reference genome assembly informed by *F*. *vesca* ssp. *bracteata*; Mb, megabase; SDR, sex-determining region.

Because *F*. *chiloensis* had the largest amount of female-specific sequence (S3 Fig) and because all SDRs from *F*. *chiloensis* females formed a monophyletic group (Fig 3), we constructed an extended SDR haplotype from female-specific sequence identified in this species. This assembly then served as a reference sequence of the full W-specific haplotype for further analyses involving the other taxa. We inferred that all female-specific sequence must be very tightly linked because—barring lethal genotype combinations, which would skew the sex ratio in ways that we do not observe [36,39]—there is no known mechanism by which multiple unlinked regions of the nuclear genome could all be female specific. This inference was validated by the constructed haplotype. Specifically, we assembled a 28 kb haplotype containing 89% of the female-specific 31-mers for this species and seven coding genes (Fig 4 and S3 Data and S3 Table). Within the full W-specific haplotype, the SDR cassette was nested within an additional 10 kb of “flanking” sequence on either side (Fig 4, middle) that included 5 kb homologous to Fvb6 position 13 Mb (split nearly evenly between left and right flanks), consistent with the SDR map location on subgenome B1 of homologous group 6 (Fig 1*B* and Table 2*A*) [40], as well as 2 kb homologous to Fvb4 position 21 Mb (right flank), accounting for the female-specific 31-mers homologous to that location (S3 Fig). These sections were nested within an additional 5 kb of “outer” sequence (Fig 4, middle) primarily showing homology to Fvb6 position 37 Mb, consistent with SDR map location on subgenome Av observed in *F*. *chiloensis* (Table 2*A*) [39]. An additional 7% of the *F*. *chiloensis* female-specific 31-mers do not align to this haplotype but are probably closely adjacent, as they also align to Fvb6 position 37 Mb. The outer section contained 31-mers that were female specific in our sample but probably not hemizygous. That is, orthologous Z pseudo-autosomal [2] sequence presumably exists with which it may potentially recombine, although ZW recombination rates are low near the *F*. *chiloensis* SDR [39]. In summary, the SDR at Fvb6 position 37 Mb encloses nested “souvenir” sequence matching the other known SDR locations (1 Mb and 13 Mb) in other taxa (Fig 1*B*), and this explains the greater proportion of female-specific 31-mers in *F*. *chiloensis* compared with the other taxa studied (S3 Fig). The female specificity of this SDR sequence, despite showing homology to disparate portions of the diploid reference genome, is consistent with movements having occurred from those locations to a new location carrying a female-determining factor.

Using the full W-specific haplotype in *F*. *chiloensis* as the reference, we characterized in detail the sequence neighboring the SDR cassette in each of the three phylogenetic clades (Fig 4). We did not assemble complete haplotypes for each clade independent of the *F*. *chiloensis* W haplotype assembly because the α clade had few female-specific 31-mers and the β clade had only three females and therefore we lacked the power to eliminate false-positive female-specific 31-mers. Instead, we identified portions of the assembled haplotype within clades that we could infer to be female specific using the following two parallel methods: alignment of female-specific 31-mers to the haplotype, and sites on the haplotype at which paired reads aligned on either side in females only (S7 Fig). These analyses revealed that distinct portions of the W haplotype were female specific in each clade (S7 Fig and S4 Table). In particular, in the α clade, only the SDR cassette is female specific. In contrast, the β clade shows female-specific sequence in both the SDR cassette and flanking sections, and the γ clade shows female-specific sequence in all three sections. The two females lacking the diagnostic deletion also did not possess any female-specific read pairs, further confirming that the SDR cassette is absent in these individuals and suggesting other mechanism(s) of male sterility [51].

The presence of sequence homologous to Fvb6 position 1 Mb within the SDR cassette in both β and γ clades (Fig 4) suggests that the SDR of the α clade and its location on Fvb6 position 1 Mb is ancestral (Fig 1*B*), with a translocation from Fvb6 position 1 Mb to position 13 Mb in the ancestor of the β and γ clades (Fig 3). A second translocation to Fvb6 position 37 Mb, specific to the γ clade (Fig 3), explains the SDR cassette and flanking sections retained in γ from its previous locations and also the outer sections unique to γ with homology to Fvb6 position 37 Mb (Fig 4), as well as the map location at Fvb6 position 37 Mb in three previously studied females in the γ clade (Fig 1*B*) [39, 43]. Therefore, based on the “souvenir” sequence that suggests that two translocations each carried adjacent sequence from their previous locations, we can propose a temporal order of SDR movements (Fig 3, black arrows).

A 2 kb portion of the downstream flanking section shows homology to Fvb4 position 21 Mb, which could be a souvenir from another prior SDR location or an independent translocation of sequence into the SDR in the β and γ ancestor; such events are commonly seen in sex chromosomes [2]. The proposed translocations must have occurred rapidly because octoploid *Fragaria* originated only approximately 1 million years ago (Mya) [46,66] and the aligned 2.7 kb portions of the SDR cassettes (Fig 2 and S2 Data) show >99% sequence similarity. This conjecture is also supported by incomplete lineage sorting of the SDR in *F*. *virginiana* (Fig 3), resulting in SDR polymorphism among females of this species. In contrast, *F*. *chiloensis*, which is monophyletic and is derived from *F*. *virginiana* ssp. *platypetala* [46], is apparently fixed for the derived SDR γ clade. All three SDR clades are found within *F*. *virginiana* ssp. *platypetala* (Fig 3), whose phylogenetic position [46,67] and geographic range (S2 Fig) lie between *F*. *virginiana* ssp. *virginiana* and *F*. *chiloensis*.

### Possible mechanisms of DNA movement

Although the mechanism of translocation of sex-determining sequence remains unknown, a striking sequence pattern suggests transposon-mediated movement. Specifically, we observe a 25 bp sequence that is inverted and repeated at the very distal ends of the flanking sections, where sequence homologous to Fvb6 13 Mb meets sequence homologous to Fvb6 37 Mb (Fig 4). On the distal end of each segment, we observe the dinucleotide motif TA. Pairs of terminal inverted repeats of 10 bp or more in length, adjacent to short duplications, are hallmarks of Class 2 transposable elements [68,69]. Therefore, this sequence signature is consistent with the hypothesis that a mobile element transported the 23 kb of SDR cassette and flanking sequence from the β clade location at Fvb6 13 Mb to the γ clade location at Fvb6 37 Mb. Terminal inverted repeats also occur in foldback elements, which can cause chromosomal rearrangement via ectopic recombination [70], and this mechanism could also facilitate movement of the SDR among homoeologs of Fvb6. We do not see terminal inverted repeats at the border between the SDR cassette and the flanking sequence, but this may have been lost, perhaps explaining why adjacent sequence was then also moved during the second translocation. Most transposable elements are under 23 kb in size, and we see no evidence of either an intact transposase, a Helitron transposon, or any known plant repetitive sequences other than stretches of dinucleotide repeats under 50 bp. Therefore, although the full W-specific haplotype remains incompletely assembled and could harbor a transposase (Fig 4), we hypothesize that the SDR movements do not involve a classic, active transposon but rather are relatively rare events that leverage active transposases that may be encoded elsewhere, as with miniature inverted-repeat transposable elements [68,69].

**Fig 5:**
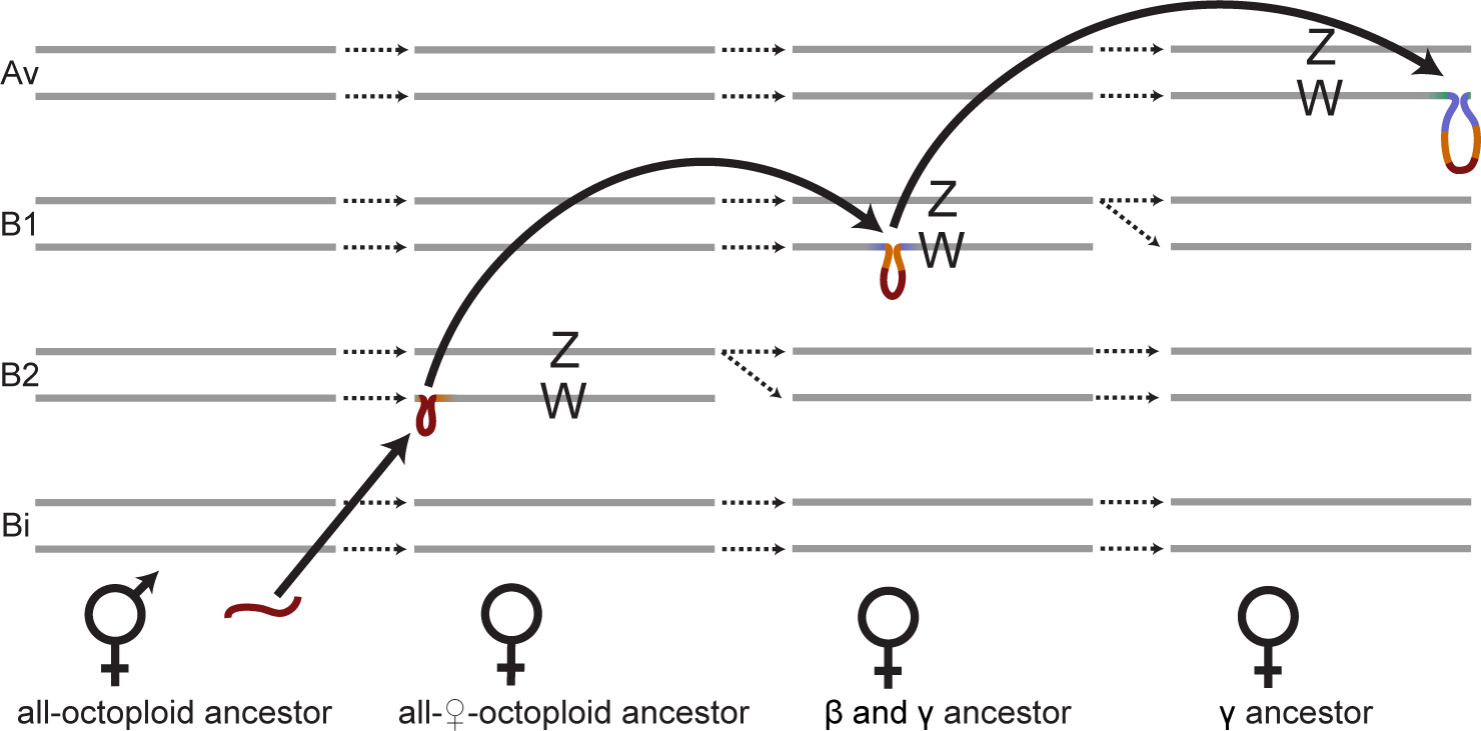
Model of sex-chromosome evolution in *Fragaria*. The eight homoeologs of Fvb6 on four subgenomes (Av, B1, B2, and Bi) are shown in a temporal sequence, starting with a presumed hermaphrodite octoploid ancestor (left). Dotted arrows indicate evolutionary descent of chromosomes. Solid arrows indicate inferred translocation or retrotransposition events. Following the move-lock-grow model, hemizygosity increases with each jump, from the retrotransposed *RPP0W* (red), to the SDR cassette including sequence homologous to the first SDR location (orange), to the SDR cassette plus flanking sequence (purple) representing the largest hemizygous region that is observed in the final SDR location. SDR, sex-determining region.

Consistent with the scenario of relatively few SDR movements, no female appears to have more than a single SDR cassette. Although we cannot assemble paralogous autosomal sequence due to high similarity among subgenomes, we can identify autosomal read pairs that align to the W haplotype but are spaced too far apart (>1 kb) to have originated in the SDR. The nonadjacent sections of the W haplotype where these paired reads align must therefore be contiguous in autosomes as they are in *Fvb*, though not in the SDR (S7 Fig and S4 Table). Coverage depth for these reads does not differ between males and females (Student *t* test, *p* > 0.1), and in females, coverage is 8-fold higher than for W-specific read pairs, suggesting that these reads originate from autosomal or pseudoautosomal regions on all four subgenomes. Therefore, there is no evidence that any autosomal homoeolog possesses an insertion representing a degraded or partial SDR. After an SDR translocation event, there would have been little or no co-occurrence of two SDR cassettes in the same female because the SDRs would occur on distinct subgenomes that segregate separately. Once separated, two SDR cassettes can never rejoin the same genome because two female plants cannot mate. Therefore, it appears that the former sex chromosomes, which have reverted to autosomes due to SDR turnover events, are descended from Z chromosomes and not W chromosomes (Fig 5).

Repeated translocation of the SDR is the only explanation consistent with all observations. Shared sequence across disparate SDRs could be explained if the shared sequences were repetitive motifs common throughout the genome, but this is not the case. Indeed, such motifs would be present in multiple copies in all individuals and thus would not be female specific. If female-specific 31-mers were false positives due to chance co-occurrence of some sequences in our female samples, we would expect to see a similar quantity of male-specific false positives, which we do not (S2 Table). Similarly, if control of sex were polygenic, then several distinct sequences could all show a correlation with sex without being physically adjacent, but this explanation can also be ruled out. Not only does sex map to a single genomic location in each of several linkage crosses [39,40,42,43,48], but under polygenic architecture, no one sequence would show a perfect correlation with sex. Furthermore, we observe sequencing reads spanning the junctions between distinct sections of the female-specific haplotype (S7 Fig), confirming that these sequences occur side by side. Therefore, the distinct sections of the SDR are adjacent only in females and must have been brought together by translocation.

### Increasing size of SDR births a new adaptive hypothesis

Chromosomal rearrangements may be especially common in polyploids [71], and because these could disrupt and/or create linkage between genes essential to sex function, they may underlie the widespread association between dioecy and polyploidy [32]. We cannot infer whether the SDR translocations have occurred at an unusually high rate relative to selectively neutral sequence. However, because rapid turnovers of SDR locations are common in evolution across many taxa [4–6,10,11], the rearrangements we observe have been plausibly favored by selection. If so, the continued coexistence of multiple SDR locations suggests that adaptive replacement may be ongoing but incomplete across these geographically widespread populations. High-density linkage maps of these octoploids [39,41] indicate conserved synteny across the homoeologs of Fvb6, with no rearrangements of Mb-sized regions, suggesting that these chromosomes have only experienced relatively small translocations. SDR translocations have been suggested to be favored by selection because this allows escape from genetic load if deleterious mutations linked to the SDR in its original location cannot be effectively purged due to lack of recombination or selective forces maintaining sex-determining alleles [13]. Alternatively, it could be advantageous for the SDR to move in order to become linked with loci under either sexually antagonistic selection [14] or other types of balancing selection without a direct connection to sex [72]. Either of these could apply in *Fragaria*. However, a third adaptive explanation is suggested by the observation that each jump of the SDR increased the size of the hemizygous female-specific haplotype by moving adjacent sequences (Figs 4 and 5 and Table 2*B*). We present this explanation as a conceptual hypothesis, but further work is required to confirm it in *Fragaria* and test it in other taxa with sex-chromosome variations.

Although we have not assembled Z chromosome sequences other than the α clade BACs, we can infer hemizygosity by assuming that the Z chromosomes have the same composition as the reference genome (Fig 4, bottom). Therefore, the SDR in the α clade is hemizygous (and shows female specificity) only for the 1.2 kb insertion containing *RPP0W*. The SDR in the β clade is hemizygous for the 13 kb SDR cassette and its two genes (*GMEW* and *RPP0W*) that show complete linkage disequilibrium with the sex-determining factor because they have no Z orthologs with which to recombine. The SDR in the γ clade is hemizygous for the 23 kb of SDR cassette and flanking sections containing five genes (*GMEW*, *RPP0W*, and three additional genes, Fig 4) in complete linkage disequilibrium with sex for the same reason. The outer sections of the γ SDR in *F*. *chiloensis* with homology to Fvb6 position 37 Mb are presumed to not be hemizygous but contain female-specific 31-mers representing variants that are in linkage disequilibrium with the hemizygous insertion. If SDR includes separate genes that are under sexually antagonistic selection—as seen for some *F*. *virginiana* traits [44]—and if two such genes are maintained polymorphic, recombination will generate maladaptive combinations [35]; a hemizygous translocated copy could thus maintain the adaptive combinations (e.g., a female-determining sequence and a female-beneficial allele of a polymorphic sexually antagonistic gene) in complete linkage disequilibrium. Therefore, translocation could represent a means of recombination suppression during sex-chromosome evolution, perhaps explaining some genomic rearrangements involving incipient SDRs and the process—“move-lock-grow”—by which the difference between the two sex chromosomes increases. *F*. *chiloensis* shows greater phenotypic differences between the sexes than other *Fragaria* species [43], as well as sex differences in recombination rates [39], and is fixed for the γ clade SDR in our samples (Fig 3 and Table 2*B*), which represents the largest hemizygous region. In contrast, *F*. *virginiana* shows less pronounced and more variable sex phenotype differentiation [44,45] and harbors SDRs from all three clades, with the α SDR being most common (Fig 3), which has the smallest hemizygous region. This is consistent with a correlation between SDR size/content and sexual dimorphism. A similar growth mechanism may underlie other hemizygous supergenes [73]. Whereas the “move-lock-grow” hypothesis is suggested by our data, future studies should test whether SDR translocations tend to increase the size of the hemizygous segment in other taxa and also whether there is an adaptive benefit to locking souvenir sequence into linkage with sex.

## Conclusion

A hemizygous SDR cassette, which contains both a gene with a known role in fruit and pollen production and a novel retrogene absent on Z and autosomal chromosomes, is conserved and has repeatedly changed genomic location across octoploid *Fragaria*, supporting a translocation model of sex-chromosome turnover (Fig 5). To our knowledge, this is the first unambiguous evidence of SDR translocation in flowering plants because it is rarely possible to distinguish translocations from de novo innovations unless putative causal sequences have been identified in more than one taxon [29,74]. In Salicaceae, SDRs occur on different chromosomes with no evidence of large-scale rearrangements, but data thus far are consistent with either master/slave regulatory dynamics [18] or SDR jumps [75,76]. Turnovers involving reversal of heterogamety, as seen in *Silene* [77], are more likely to be fusions of sex chromosomes to autosomes rather than translocations of SDR sequence to new chromosomes. Our discovery of a conserved yet mobile W-specific SDR helps to unify extensive and disparate research on the genetic basis of dioecy in *Fragaria* and across flowering plants [41,74]. It suggests that independent mechanisms of dioecy within closely related taxa may be rarer than they appear. Instead, SDR translocation can maintain the same genetic basis for sex while adjusting genomic location and accumulating sequence that may contain sexually antagonistic alleles as well as increasing recombination suppression within the growing hemizygous SDR. The “move-lock-grow” phenomenon may allow for rapid and extensive change in sex chromosomes, perhaps influencing sexual dimorphism, hybrid compatibility, recombination rates, or other traits of evolutionary or ecological importance.

## Materials and methods

### Sex phenotyping

We determined sex using our established method [51]. In brief, we grew plants with 513 mg granular Nutricote 13:13:13 N:P:K fertilizer (Chisso-Asahi Fertilizer) under 15:20°C night:day temperatures and 10 to 12-hour days and then exposed them to 8:12°C night:day temperatures with an 8-hour low-light day to initiate flowering. Fertilizer and pest control measures were applied as needed. Male function was scored as a binary trait: plants with large, bright-yellow anthers that visibly released pollen were “male (male-fertile),” and plants with vestigial white or small, pale-yellow anthers that neither dehisced nor showed mature pollen were “female (male-sterile).” Because of the tight correlation between male function and female function, male sterility serves as a good phenotypic marker of the SDR [39].

### DNA extraction and quantification

Genomic DNA was extracted from silica dried leaf tissues using Norgen Biotek Plant/Fungi DNA Isolation 96-Well Kit (Ontario, Canada) and by the service provider Ag-Biotech (Monterey, CA). An additional 100 μl 10% SDS and 10 μl β-mercaptoethanol were added to the lysis buffer to improve DNA yield. DNA was further purified with sodium acetate and ethanol precipitation. DNA concentration was quantified by Quant-iT PicoGreen (Invitrogen, Carlsbad, CA) assays at University of Pittsburgh Genomics and Proteomics Core Laboratories (GPCL).

### Whole-genome sequencing

For the whole-genome analysis, we examined 60 outbred, unrelated plants distributed across the geographic ranges of the octoploid *Fragaria* species (S1 Table). These samples were collected from the wild as clones or obtained from the USDA National Clonal Germplasm Repository. Genomic DNA extraction and library preparation were performed by the Oregon State University Center for Genome Research and Biocomputing (CGRB) and at University of Pittsburgh. We sheared DNA to 300 bp using a Bioruptor Pico (Diagenode, Denville, NJ) and used the NEBNext Ultra DNA Library Prep Kit for Illumina (New England BioLabs, Ipswich, MA) with individually indexed dual barcodes. We sequenced whole genomes of 60 *Fragaria* samples using four lanes of paired-end 150 bp on an Illumina HiSeq 3000, with 13 to 20 samples per lane (Table 1). Although reads were not aligned to the diploid *F*. *vesca* reference genome *Fvb*, we report coverage relative to this reference as the sum of lengths of all sequenced reads divided by the size of *Fvb* (e.g., 8× coverage relative to *Fvb* should mean approximately 1× coverage per chromosome in an octoploid).

We converted FASTQ files to FASTA and used Jellyfish 1.0.2 [78] to count 31-mers in each sample, the largest k-mer size allowed by Jellyfish (S1 Fig). We used the Linux “sort” and “join” functions to combine lists of 31-mers and generate lists of 31-mers shared by sets of females (defined taxonomically or as α, β+γ, or γ clade) and absent in all male plants. As a control to ensure this method was not yielding false positives or repetitive sequence (e.g., from heterochromatin), we also searched for male-specific 31-mers, which are not expected to exist because the Z chromosome is present in both sexes. As with the females, we searched for male-specific 31-mers within clades (for males, defined as the clade of the closest-related female plant, as determined by chloroplast phylogeny [46]). To aid the assembly of the full W-specific haplotype in *F*. *chiloensis*, we also generated lists of 31-mers that were female specific in that species (ignoring males of other species), as well as 31-mers shared in all but one female, assuming that a W-specific 31-mer could be absent due to insufficient coverage or a rare sequence variant. The assembly was feasible because these nearly female-specific 31-mers were densely spaced across the SDR haplotype, such that median span between nonadjacent 31-mers was 100 bp, and 90% of them were separated by less than 500 bp (excluding the two unassembled gaps), typically within the range spanned by paired-end reads. We aligned these 31-mers to *Fvb* using BLAT version 32x1 [79] and retained hits with at least 29 bp matching and gaps no larger than 30 bp. We extracted reads containing female-specific 31-mers and their mate pairs from the original FASTQ files. We assembled these manually in BioEdit version 7.2.5 [80], beginning at the diagnostic deletion and moving outward in both directions, when possible guiding the assembly with alignment to homologous *Fvb* or BAC sequences. Gaps between contigs containing female-specific 31-mers were manually joined with additional reads as possible. We assembled the central 2.7 kb of the SDR cassette, including the diagnostic deletion and *RPP0W*, for all females possessing it. We assembled a pseudo-outgroup sequence based on homologous portions of *Fvb* and BAC6 and used RAxML [81] with -m GTRCAT to generate a phylogeny of the W sequence. Major clades (α, β, and γ) were assigned visually. We used a consensus sequence of *RPP0W* (966 bp) from each of the three clades to generate a phylogeny with the four *RPP0W* paralogs in *Fvb*, again using RAxML [81] with -m GTRCAT.

We assigned portions of the W haplotype to *Fvb* regions using BLAST at GDR [82] (S3 Table). We identified genes using GENSCAN [83] and annotated them with BLAST to the NCBI database and to *Fvb—*which is annotated—using GDR [82]. Adjacent genes (S5 Table) were identified from the *Fvb* annotation. Gene expression data in *F*. *vesca* [63] were extracted from http://mb3.towson.edu/efp/cgi-bin/efpWeb.cgi. We looked for significant (E-value < 0.05) hits to repetitive sequence by BLASTing to the TIGR Plant Repeat Databases [84] with GDR [82]. We search for Helitron transposons using HelitronScanner [85].

### BAC sequencing and amplicon fine-mapping

A BAC library was prepared by Chris Saski, Clemson University Genomics Institute (CUGI) from 90 g leaf tissue collected at the University of Pittsburgh from Y33b2, the female parent of the *F*. *virginiana* ssp. *virginiana* linkage-mapping cross [42,51]. BAC construction methods followed Luo and Wing [86], with minor modifications. We designed overgo probes from the mapped male sterility region between Fvb6 positions 1.626 Mb and 1.794 Mb (S6 Table). We labeled probes individually with 32_P following the CUGI protocol (http://www.genome.clemson.edu/resources/protocols) and hybridized them to the BAC filters at 60°C overnight. This yielded 69 positive clones (S6 Table).

Genomic libraries from these 69 BACs were individually prepared and barcode indexed with the Illumina TruSeq DNA HT kit and sequenced with 150 bp paired-end reads on a single lane of Illumina MiSeq at Oregon State University CGRB. Reads were quality trimmed for both Q > 20 and Q > 30 with Trimmomatic [87] and merged, when possible, with the program FLASH [88]. We filtered merged reads and unmerged pairs by digital normalization at coverage of 100 using khmer [89]. For each library, both quality trimming sets were de novo assembled with Velvet [90] using a range of kmers from 31 to 91 bp. We selected the assembly with the longest contig for downstream analyses of each BAC (S6 Table).

We masked vectors with bedtools [91] and used BLAT to identify identical overlap of >1 kb among BACs. Groups of BACs representing putative homoeologs were imported into Geneious R7 [92] and further scaffolded manually. The resulting 11 assemblies were assigned to homoeologs (S4 Fig) by the presence of linkage-mapped SNPs observed in the target capture and microfluidic markers. We MAFFT [93]-aligned eight scaffolds (excluding non-overlapping scaffolds 6, 9, and 10) with a mean length of 47.993 kb. We removed all gap positions, resulting in a 19.819 kb alignment. We estimated a maximum likelihood tree with PhyML [94], confirming the identification of four pairs of homologous chromosomes (S5 Fig and S4 Data).

F1 offspring from the previously described *F*. *virginiana* ssp. *virginiana* cross “Y33b2×O477” [42,51] were sexed (*N* = 1,878) as described above and genotyped (*N* = 184) at sex-linked microsatellite markers [42] to identify possible recombinants, which were sequenced with targeted capture (*N* = 67) as previously described [41]. We designed Fluidigm microfluidic markers for fine-mapping Y33b2×O477 following our previous methodology [39]. We designed primer pairs for 48 amplicons with mean expected size of 385 bp—12 and 16 on the two BAC contigs corresponding to the Z homoeolog (S4 Fig) and 20 between Fvb6 positions 0.716 Mb to 17.605 Mb (S7 Table). We used the Fluidigm 48.48 Access Array Integrated Fluidic Circuits (IFCs) at the University of Idaho IBEST for amplicon library preparation following standard simplex reaction protocol. We pooled the amplicons of 190 F1 offspring and the two parents for paired-end 300 bp sequencing on a one-quarter lane of Illumina MiSeq. We trimmed reads as above, aligned them to *Fvb* and the BAC sequences using BWA version 0.7.12 [95], and called genotypes with POLiMAPS [41]. We identified recombinants and used these to define the narrowest possible window overlapping male function.

## Supporting information

S1 Fig Schematic of our analysis pipeline. Blue boxes represent data files. Pink boxes represent analytical steps.
(PDF)

S2 Fig Collection localities and SDR map locations for all samples collected from five North American taxa. See Tables 1 and S1 for details. SDR, sex-determining region.
(PDF)

S3 Fig Female-specific 31-mers with sequence similarity to *Fvb* reference genome. These 31-mers do not match the reference genome perfectly but show where there is homology with different portions of the reference genome, indicating the likely evolutionary origins of female-specific sequence. Boxes are colored according to sequence similarity with *Fvb*, or as overlapping the diagnostic deletion (with homology to Fvb6 position 1.636 Mb). Sequences (31-mers) aligning to all three SDR map locations are observed, though not in all groups. Counts of 31-mers are indicated on the y-axis (each 31-mer is only counted once per group, regardless of sequencing depth). 31-mers not aligning to *Fvb* are not shown (S2 Table). All females share sequence homologous to Fvb6 position 1 Mb and Fvb7 position 18 Mb. (A) Organized by taxonomy. (B) Organized by clade (α, β, and/or γ; Fig 3) within *F*. *virginiana* samples. Note that β clade alone is not shown because of insufficient sample size (two females). The α clade female-specific 31-mers align only to its map location at Fvb6 position 1 Mb but not the other map locations. In contrast, *F*. *virginiana* β and γ clades both possess female-specific 31-mers aligning to Fvb6 positions 1 Mb and 13 Mb, and only the *F*. *virginiana* γ clade possesses female-specific 31-mers aligning to Fvb6 position 37 Mb, mirroring the results for *F*. *chiloensis*, which is also in the γ clade (part A). SDR, sex-determining region.
(PDF)

S4 Fig Sequenced BAC clones aligned between Fvb6 positions 1.624 Mb and 1.798 Mb. Using offspring of the *Fragaria v*. ssp. *virginiana* cross [42], we fine-mapped this SDR (previously determined to be on subgenome B2 within Fvb6 range 0–5.5 Mb) to a 140 kb region between positions 1.630 Mb and 1.770 Mb on Fvb6, using methods similar to those that localized the two other SDRs (Tables 1 and 2*A*) [39,40]. We sequenced and assembled 62 maternal-parent BACs overlapping this 140 kb region. BACs were identified with four overgo probes (S6 Table). BAC clones are assembled by color into inferred contigs, labeled according to subgenome (Av, B1, B2, or Bi) and an arbitrary number (“Scaffold Group” in S6 Table). Scale bar in kb indicated in lower right. The subgenome B2 contigs are designated as “r” (“in repulsion,” i.e., the Z chromosome) or “c” (“in coupling,” i.e., the W chromosome). Fluidigm probes were designed from BAC contigs 6 and 8 corresponding to the Z chromosome (S6 Table). Scaffold groups 7 and 9 are presumed to represent the same chromosome, but a single assembly integrating the two was not achieved. Scaffold group 10 could not be assigned to subgenome and is not depicted. Outside of subgenome B2, BACs are not depicted if completely redundant with another BAC. Note that no portion of the W chromosome was recovered from the male-sterility region fine-mapped between Fvb6 position 1.630 Mb and Fvb6 position 1.770 Mb (top; region with red genes from gene16560 to gene16538). Gene16559, the Fvb6 homolog of *GMEW*, is highlighted in yellow. BAC, bacterial artificial chromosome; SDR, sex-determining region.
(PDF)

S5 Fig Phylogeny of assembled BAC sequences. Across a 19.8 kb alignment, BAC scaffold groups form four distinct and well-supported clades, corresponding to the four subgenomes. Numbers on branches are bootstraps. BAC scaffold groups that did not overlap this alignment region are not shown (S4 Fig and S6 Table). BAC, bacterial artificial chromosome.
(PDF)

S6 Fig Phylogeny of *RPP0W* and its autosomal paralogs. We aligned consensus sequences of *RPP0W* (Fig 2) from the three SDR clades (α, β, and γ, Fig 3) with the four paralogous genes from the *Fvb* diploid reference genome. Bootstrap support is indicated above the branches. The most closely related genes are on Fvb5 and Fvb7, explaining the female-specific 31-mers that align to these chromosomes (S3 Fig). *RPP0W* sequences across SDR clades form a monophyletic group, consistent with a single origin. SDR, sex-determining region.
(PDF)

S7 Fig Autosomal paired-end reads and female-specific 31-mers aligned to W haplotype. Color-coding of haplotype follow Fig 4. Female-specific 31-mers in each of the three clades (α, β, and γ) are aligned to the assembled haplotype. Sites on the haplotype that were spanned by paired reads females but never in males (“seams,” white boxes) represent pairs of sequences that are directly adjacent only on the W chromosome at the SDR, although they may occur individually elsewhere in the genome (S4 Table). The distribution of these seams among clades parallels the distribution of female-specific 31-mers; one seam present in α and γ but not β may be missing by chance in our data due to low β sample size (S4 Table). Nonadjacent sequence immediately outside of the three insertion sites (Fig 4) is spanned by a large number of read pairs in all samples regardless of sex. This suggests that these sequences, which are adjacent in the *Fvb* reference genome, are also adjacent in autosomal and Z-specific paralogs, probably across all four subgenomes because coverage is 8-fold higher than for W-specific read pairs. We see no evidence of any partial or pseudogenized W haplotype at these autosomal locations. SDR, sex-determining region.
(PDF)

S1 Table Unrelated plants examined with whole-genome sequencing. (DOCX)

S2 Table Sex-specific 31-mers per group. (DOCX)

S3 Table Female-specific sequence. (DOCX)

S4 Table Read pairs aligned to key regions of W haplotype. (DOCX)

S5 Table Genes within 50 kb of the three SDR locations, as per *Fragaria vesca* reference genome *Fvb*. SDR, sex-determining region. (DOCX)

S6 Table BAC sequencing and assembly data. BAC, bacterial artificial chromosome. (DOCX)

S7 Table Primers for amplicon sequencing. (DOCX)

S1 Data Alignment of *RPP0W* and homologs used for phylogenetic analysis (S6 Fig). (FAS)

S2 Data Alignment of W-specific haplotype sequences used for phylogenetic analysis (Fig 3). (FAS)

S3 Data Assembled SDR haplotype from *F*. *chiloensis*. (FAS)

S4 Data Alignment of BAC sequences used for phylogenetic analysis (S5 Fig). (FAS)

## Abbreviations

BAC: bacterial artificial chromosome
CGRB: Center for Genome Research and Biocomputing
CUGI: Clemson University Genomics Institute
Fvb: diploid reference genome assembly informed by *F*. *vesca* ssp. *bracteata*
GPCL: Genomics and Proteomics Core Laboratories
IFC: Integrated Fluidic Circuit
Mb: megabase
Mya: million years ago
QTL: quantitative trait locus
SDR: sex-determining region
