# Supplementary figures and images for: Repeated translocation of a gene cassette drives sex-chromosome turnover in strawberries
Source: PLoS Biol. 2018 Aug 27;16(8):e2006062. doi: 10.1371/journal.pbio.2006062 (PMC6128632; doi:10.1371/journal.pbio.2006062)

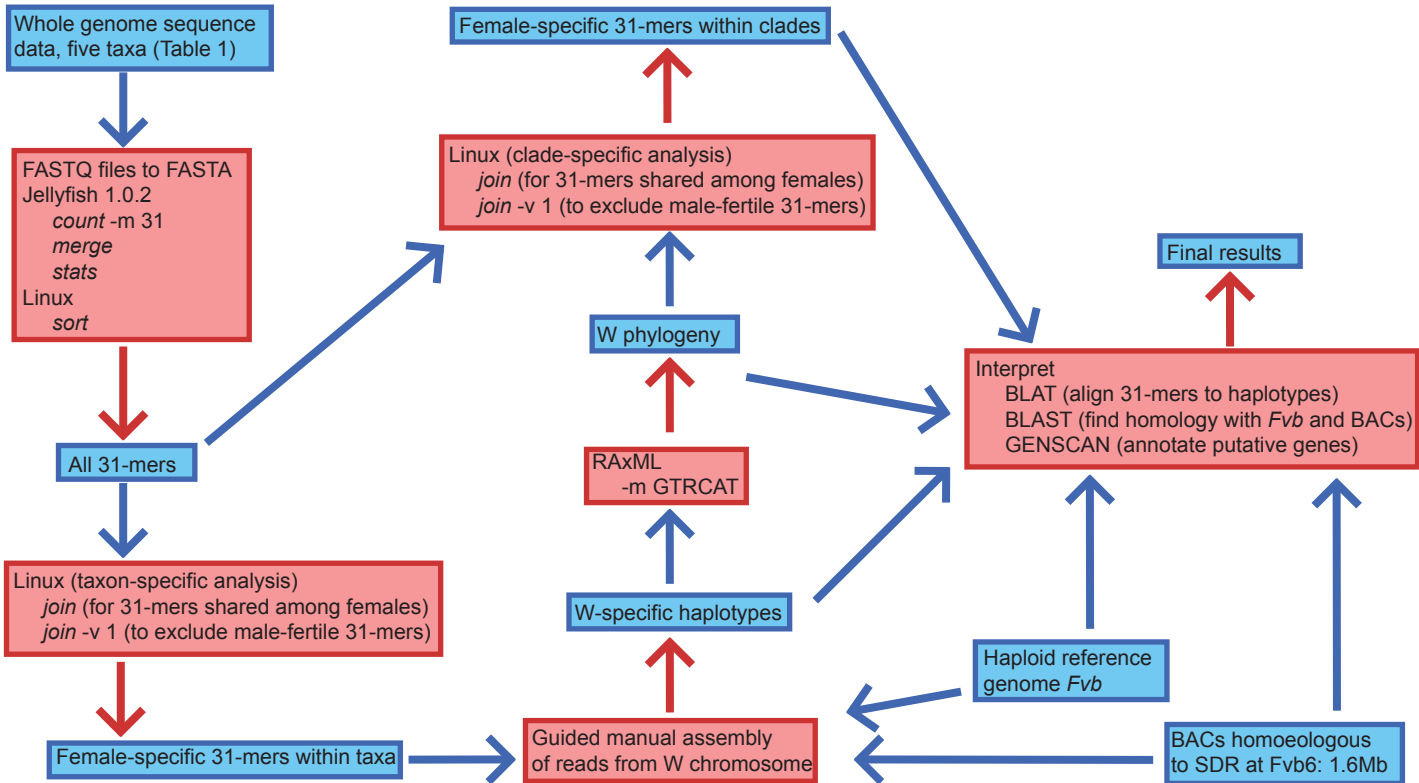

Supplement: S1 Fig — Pink boxes represent analytical steps. (PDF) [file pbio.2006062.s001.pdf]

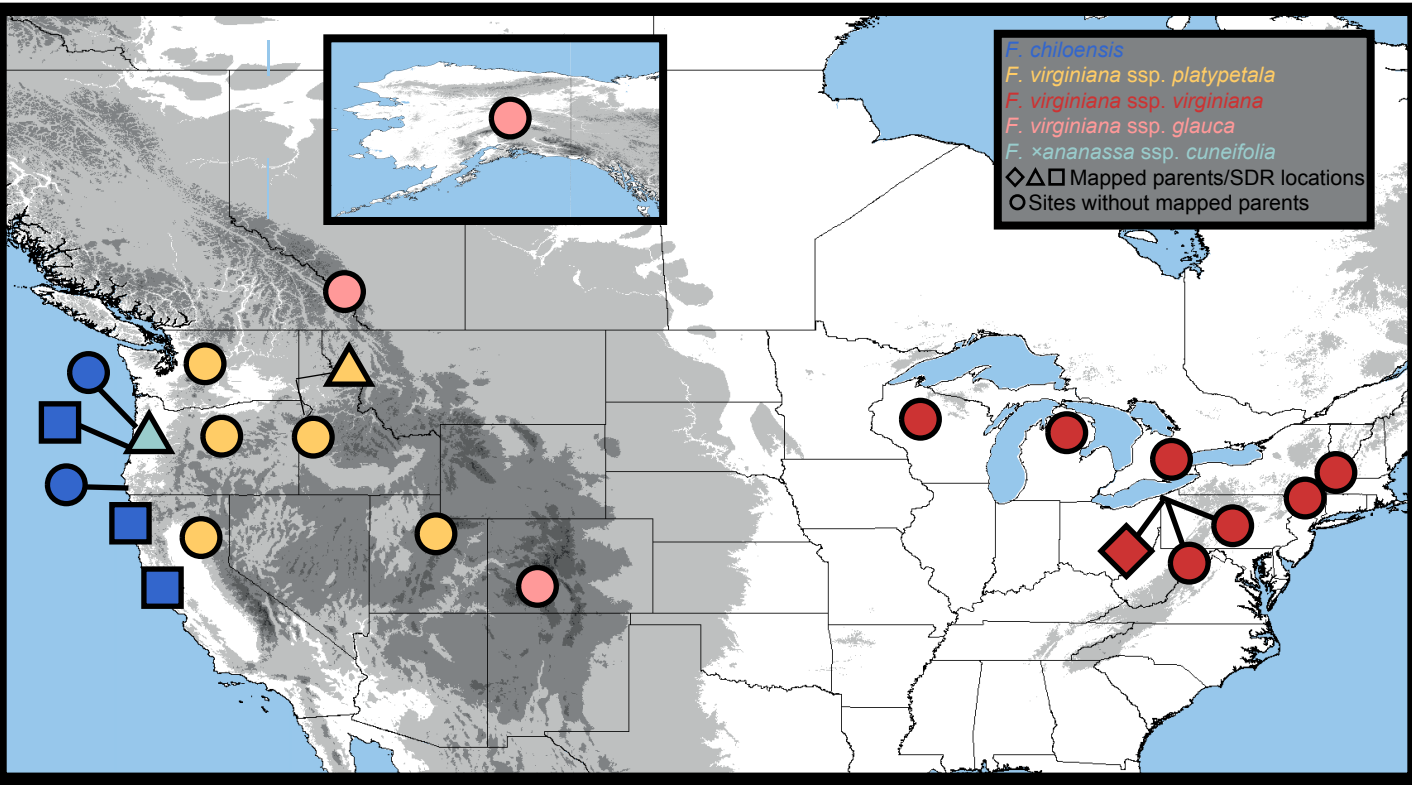

Supplement: S2 Fig — See Tables 1 and S1 for details. SDR, sex-determining region. (PDF) [file pbio.2006062.s002.pdf]

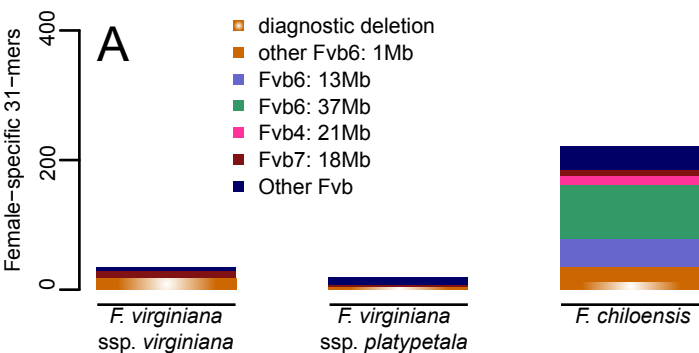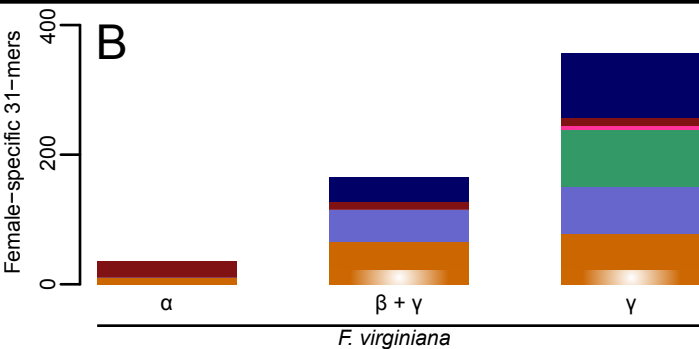

Supplement: S3 Fig — These 31-mers do not match the reference genome perfectly but show where there is homology with different portions of the reference genome, indicating the likely evolutionary origins of female-specific sequence. Boxes are colored according to sequence similarity with Fvb, or as overlapping the diagnostic deletion (with homology to Fvb6 position 1.636 Mb). Sequences (31-mers) aligning to all three SDR map locations are observed, though not in all groups. Counts of 31-mers are indicated on the y-axis (each 31-mer is only counted once per group, regardless of sequencing depth). 31-mers not aligning to Fvb are not shown (S2 Table). All females share sequence homologous to Fvb6 position 1 Mb and Fvb7 position 18 Mb. (A) Organized by taxonomy. (B) Organized by clade (α, β, and/or γ; Fig 3) within F. virginiana samples. Note that β clade alone is not shown because of insufficient sample size (two females). The α clade female-specific 31-mers align only to its map location at Fvb6 position 1 Mb but not the other map locations. In contrast, F. virginiana β and γ clades both possess female-specific 31-mers aligning to Fvb6 positions 1 Mb and 13 Mb, and only the F. virginiana γ clade possesses female-specific 31-mers aligning to Fvb6 position 37 Mb, mirroring the results for F. chiloensis, which is also in the γ clade (part A). SDR, sex-determining region. (PDF) [file pbio.2006062.s003.pdf]

gene16661-16660-16661 in B2 only

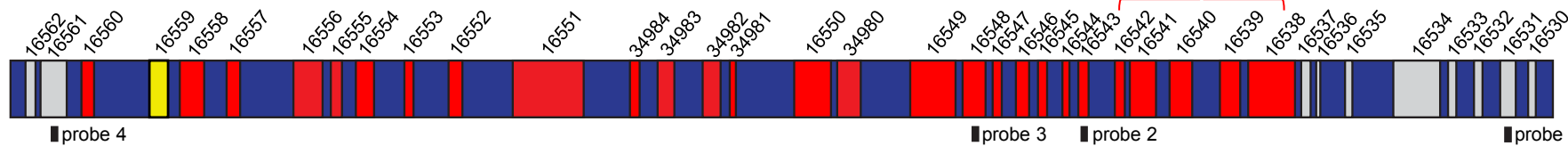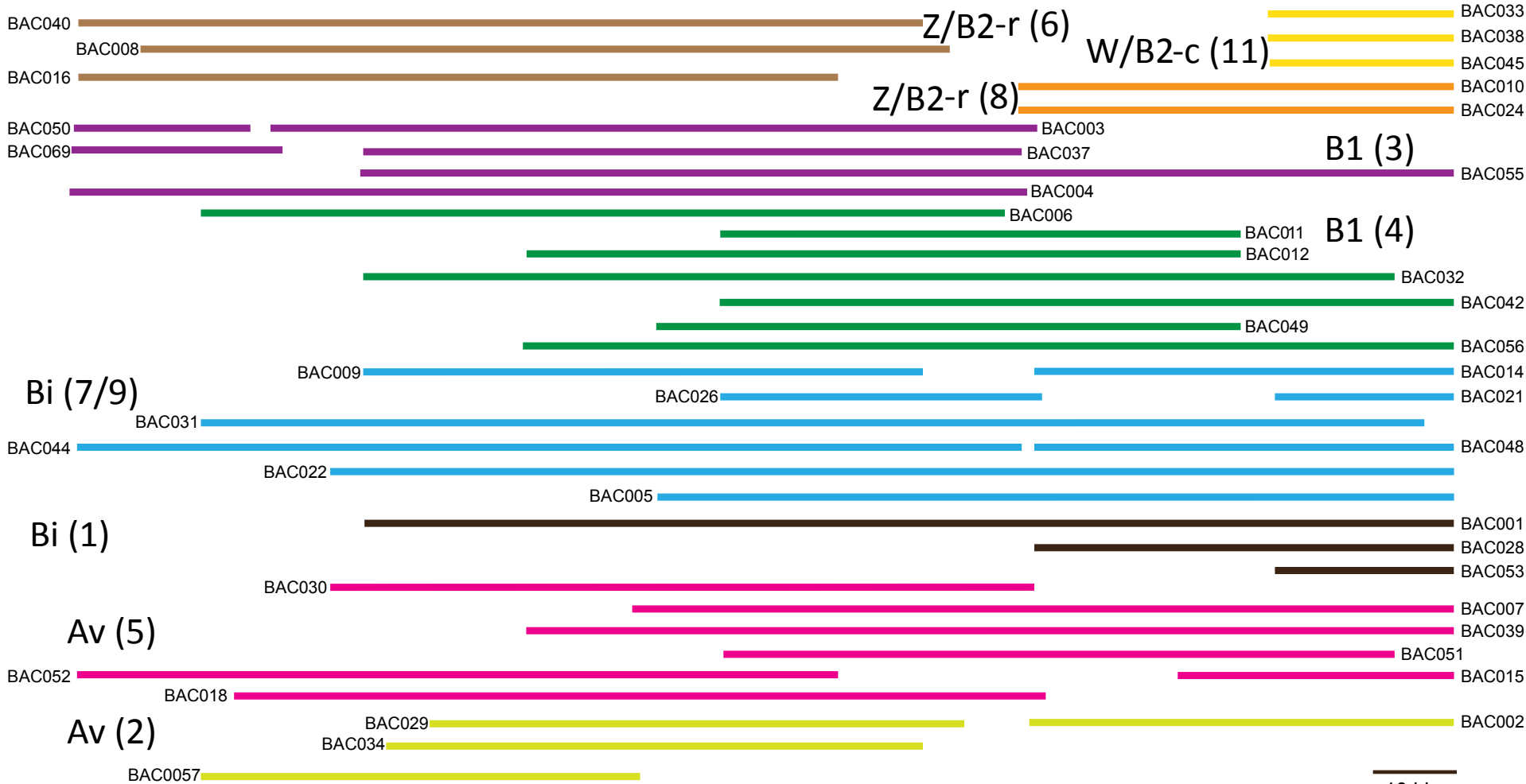

Supplement: S4 Fig — Using offspring of the Fragaria v. ssp. virginiana cross [42], we fine-mapped this SDR (previously determined to be on subgenome B2 within Fvb6 range 0–5.5 Mb) to a 140 kb region between positions 1.630 Mb and 1.770 Mb on Fvb6, using methods similar to those that localized the two other SDRs (Tables 1 and 2A) [39,40]. We sequenced and assembled 62 maternal-parent BACs overlapping this 140 kb region. BACs were identified with four overgo probes (S6 Table). BAC clones are assembled by color into inferred contigs, labeled according to subgenome (Av, B1, B2, or Bi) and an arbitrary number (“Scaffold Group” in S6 Table). Scale bar in kb indicated in lower right. The subgenome B2 contigs are designated as “r” (“in repulsion,” i.e., the Z chromosome) or “c” (“in coupling,” i.e., the W chromosome). Fluidigm probes were designed from BAC contigs 6 and 8 corresponding to the Z chromosome (S6 Table). Scaffold groups 7 and 9 are presumed to represent the same chromosome, but a single assembly integrating the two was not achieved. Scaffold group 10 could not be assigned to subgenome and is not depicted. Outside of subgenome B2, BACs are not depicted if completely redundant with another BAC. Note that no portion of the W chromosome was recovered from the male-sterility region fine-mapped between Fvb6 position 1.630 Mb and Fvb6 position 1.770 Mb (top; region with red genes from gene16560 to gene16538). Gene16559, the Fvb6 homolog of GMEW, is highlighted in yellow. BAC, bacterial artificial chromosome; SDR, sex-determining region. (PDF) [file pbio.2006062.s004.pdf]

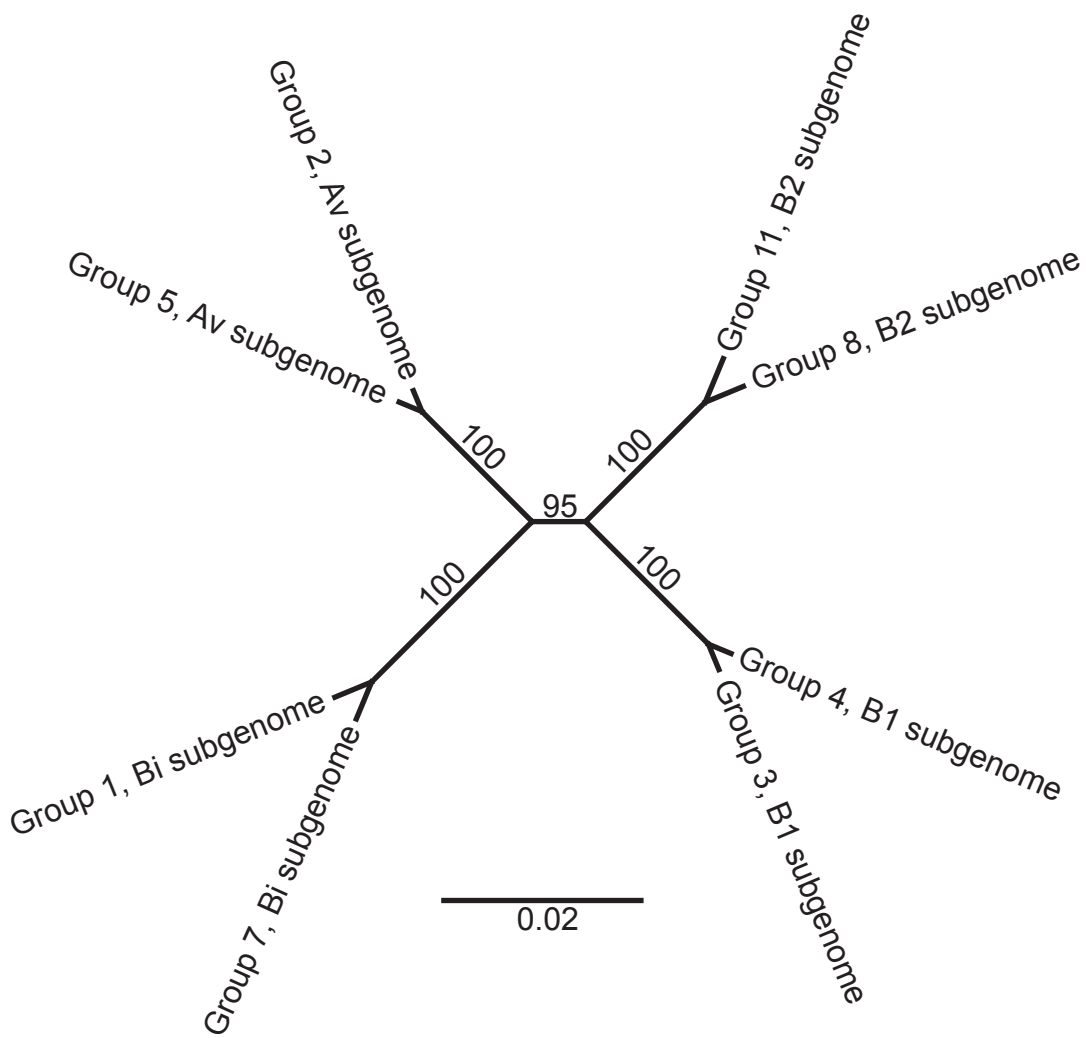

Supplement: S5 Fig — Across a 19.8 kb alignment, BAC scaffold groups form four distinct and well-supported clades, corresponding to the four subgenomes. Numbers on branches are bootstraps. BAC scaffold groups that did not overlap this alignment region are not shown (S4 Fig and S6 Table). BAC, bacterial artificial chromosome. (PDF) [file pbio.2006062.s005.pdf]

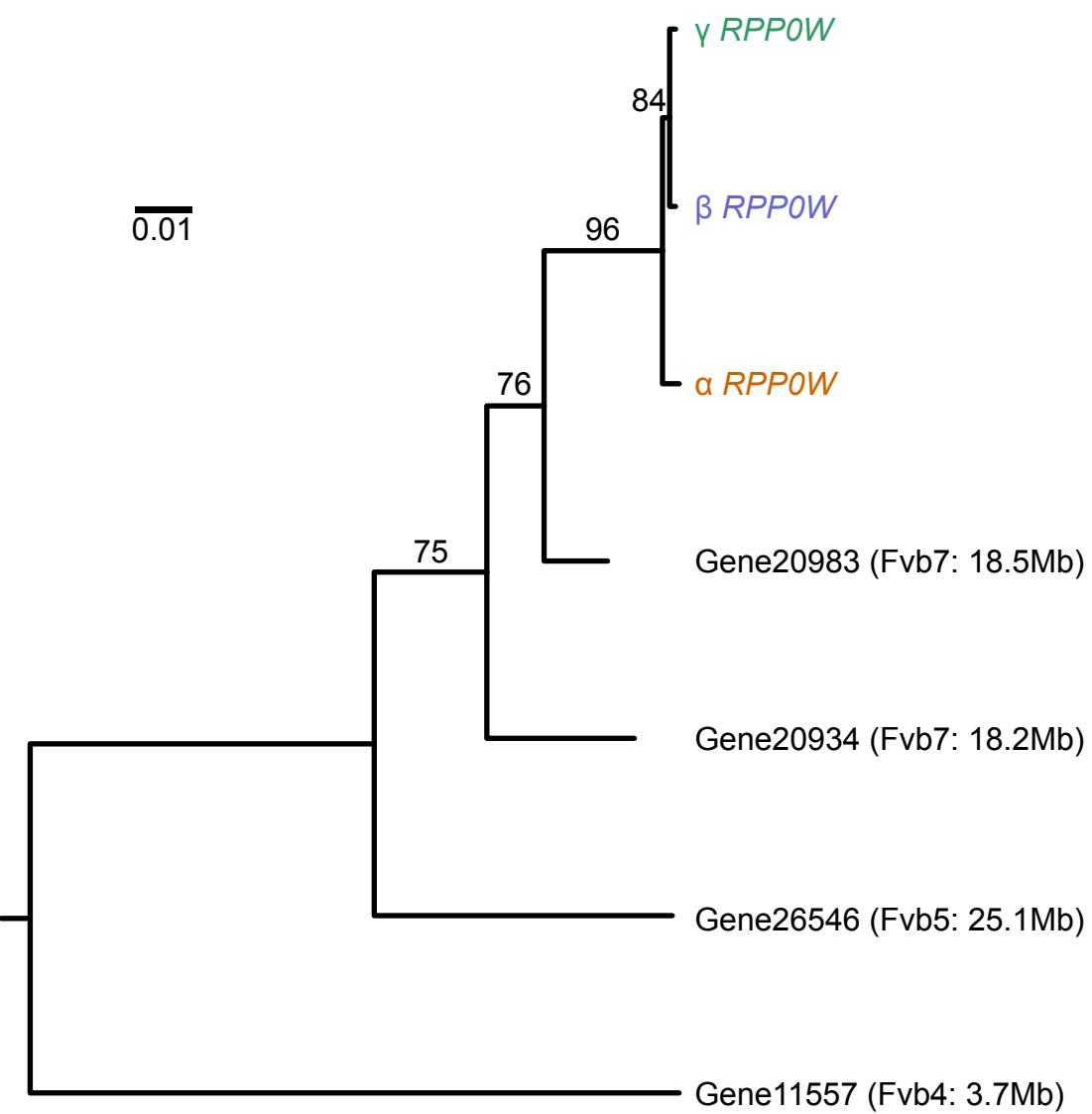

Supplement: S6 Fig — We aligned consensus sequences of RPP0W (Fig 2) from the three SDR clades (α, β, and γ, Fig 3) with the four paralogous genes from the Fvb diploid reference genome. Bootstrap support is indicated above the branches. The most closely related genes are on Fvb5 and Fvb7, explaining the female-specific 31-mers that align to these chromosomes (S3 Fig). RPP0W sequences across SDR clades form a monophyletic group, consistent with a single origin. SDR, sex-determining region. (PDF) [file pbio.2006062.s006.pdf]

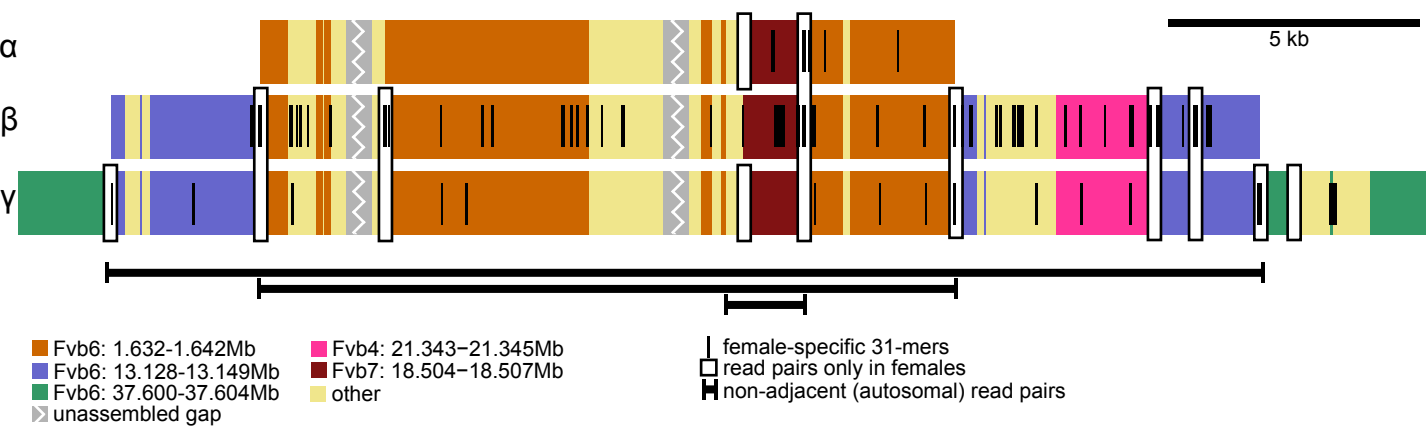

Supplement: S7 Fig — Color-coding of haplotype follow Fig 4. Female-specific 31-mers in each of the three clades (α, β, and γ) are aligned to the assembled haplotype. Sites on the haplotype that were spanned by paired reads females but never in males (“seams,” white boxes) represent pairs of sequences that are directly adjacent only on the W chromosome at the SDR, although they may occur individually elsewhere in the genome (S4 Table). The distribution of these seams among clades parallels the distribution of female-specific 31-mers; one seam present in α and γ but not β may be missing by chance in our data due to low β sample size (S4 Table). Nonadjacent sequence immediately outside of the three insertion sites (Fig 4) is spanned by a large number of read pairs in all samples regardless of sex. This suggests that these sequences, which are adjacent in the Fvb reference genome, are also adjacent in autosomal and Z-specific paralogs, probably across all four subgenomes because coverage is 8-fold higher than for W-specific read pairs. We see no evidence of any partial or pseudogenized W haplotype at these autosomal locations. SDR, sex-determining region. (PDF) [file pbio.2006062.s007.pdf]
