# Supplementary material for: Repeated translocation of a gene cassette drives sex-chromosome turnover in strawberries
Source: PLoS Biol. 2018 Aug 27;16(8):e2006062. doi: 10.1371/journal.pbio.2006062 (PMC6128632; doi:10.1371/journal.pbio.2006062)
Supplement: S1 Table — (DOCX) [file pbio.2006062.s008.docx]

**S1 Table. Unrelated plants examined with whole genome sequencing.**

| **Name** | **Sex** | **Diagnostic deletion** | **Clade^a^** | **Parent in Map Cross (Citation)** | **Location** | **Taxon** | **NCBI SRA Accession** |
| --- | --- | --- | --- | --- | --- | --- | --- |
| WREN7 | Female (male-sterile) | Yes | β | Govindarajulu et al. 2013 | Wren, OR | *F*. × *ananassa* ssp. *cuneifolia* | SRR6149701 |
| MP10 | Male-fertile | No | γ | Govindarajulu et al. 2013 | Mary's Peak, OR | *F*. × *ananassa* ssp. *cuneifolia* | SRR6149723 |
| WREN2 | Male-fertile | No | n/a | Govindarajulu et al. 2013 | Wren, OR | *F*. × *ananassa* ssp. *cuneifolia* | SRR6149708 |
| EUR12.1 | Female (male-sterile) | Yes | γ | No | Eureka, CA | *F. chiloensis* ssp. *pacifica* | SRR6149676 |
| EUR13.2 | Female (male-sterile) | Yes | γ | Tennessen et al. 2016 | Eureka, CA | *F. chiloensis* ssp. *pacifica* | SRR6149675 |
| EUR17.1 | Female (male-sterile) | Yes | γ | No | Eureka, CA | *F. chiloensis* ssp. *pacifica* | SRR6149682 |
| GP33.1/HM1.1 | Female (male-sterile) | Yes | γ | Goldberg et al. 2010 | Honeyman State Park, OR | *F. chiloensis* ssp. *pacifica* | SRR6149717 |
| HM12.1 | Female (male-sterile) | Yes | γ | No | Honeyman State Park, OR | *F. chiloensis* ssp. *pacifica* | SRR6149716 |
| HM16.1 | Female (male-sterile) | Yes | γ | No | Honeyman State Park, OR | *F. chiloensis* ssp. *pacifica* | SRR6149718 |
| PTR14.3 | Female (male-sterile) | Yes | γ | Tennessen et al. 2016 | Point Reyes, CA | *F. chiloensis* ssp. *pacifica* | SRR6149705 |
| SAL6.1 | Female (male-sterile) | Yes | γ | No | Glen Eden Beach, OR | *F. chiloensis* ssp. *pacifica* | SRR6149704 |
| SAL8.1 | Female (male-sterile) | Yes | γ | No | Glen Eden Beach, OR | *F. chiloensis* ssp. *pacifica* | SRR6149710 |
| EUR3.4 | Male-fertile | No | γ | Tennessen et al. 2016 | Eureka, CA | *F. chiloensis* ssp. *pacifica* | SRR6149680 |
| EUR2.1 | Male-fertile | No | γ | No | Eureka, CA | *F. chiloensis* ssp. *pacifica* | SRR6149681 |
| EUR9.1 | Male-fertile | No | γ | No | Eureka, CA | *F. chiloensis* ssp. *pacifica* | SRR6149679 |
| HM13.1 | Male-fertile | No | γ | No | Honeyman State Park, OR | *F. chiloensis* ssp. *pacifica* | SRR6149719 |
| PIS3.1 | Male-fertile | No | γ | No | Pistol River, OR | *F. chiloensis* ssp. *pacifica* | SRR6149694 |
| PIS5.1 | Male-fertile | No | γ | No | Pistol River, OR | *F. chiloensis* ssp. *pacifica* | SRR6149702 |
| PTR5.3 | Male-fertile | No | γ | No | Point Reyes, CA | *F. chiloensis* ssp. *pacifica* | SRR6149703 |
| PTR17.1 | Male-fertile | No | γ | No | Point Reyes, CA | *F. chiloensis* ssp. *pacifica* | SRR6149672 |
| PTR19.2 | Male-fertile | No | γ | Tennessen et al. 2016 | Point Reyes, CA | *F. chiloensis* ssp. *pacifica* | SRR6149713 |
| SAL3.1 | Male-fertile | No | γ | Goldberg et al. 2010 | Glen Eden Beach, OR | *F. chiloensis* ssp. *pacifica* | SRR6149684 |
| SAL4.1 | Male-fertile | No | γ | No | Glen Eden Beach, OR | *F. chiloensis* ssp. *pacifica* | SRR6149673 |
| NA.AB.2.7.3 | Female (male-sterile) | No | n/a | No | Upper Kananaskis Lake, AB | *F. virginiana* ssp. *glauca* | SRR6149722 |
| NA.AK.4.14.3 | Female (male-sterile) | Yes | α | No | Chena River, AK | *F. virginiana* ssp. *glauca* | SRR6149689 |
| NA.CO.1.6.3 | Female (male-sterile) | No | n/a | No | Los Pinos Creek, CO | *F. virginiana* ssp. *glauca* | SRR6149685 |
| GH5.2 | Female (male-sterile) | Yes | β | No | Rose Creek, WA | *F. virginiana* ssp. *platypetala* | SRR6149714 |
| KB3 | Female (male-sterile) | Yes | β | Wei et al. in press | Kamiak Butte, WA | *F. virginiana* ssp. *platypetala* | SRR6149720 |
| NA.CA.12.7.4 | Female (male-sterile) | Yes | γ | No | Longville, CA | *F. virginiana* ssp. *platypetala* | SRR6149692 |
| NA.OR.3.4.3 | Female (male-sterile) | Yes | α | No | Walton Lake, OR | *F. virginiana* ssp. *platypetala* | SRR6149707 |
| NA.OR.3.6.4 | Female (male-sterile) | Yes | γ | No | Walton Lake, OR | *F. virginiana* ssp. *platypetala* | SRR6149706 |
| NA.UT.2.12.3 | Female (male-sterile) | Yes | γ | No | Lodgepole Campground, UT | *F. virginiana* ssp. *platypetala* | SRR6149728 |
| NA.UT.2.20.3 | Female (male-sterile) | Yes | γ | No | Lodgepole Campground, UT | *F. virginiana* ssp. *platypetala* | SRR6149727 |
| NA.WA.1.13.3 | Female (male-sterile) | Yes | α | No | Tucquala Lake, WA | *F. virginiana* ssp. *platypetala* | SRR6149699 |
| NA.WA.1.2.4 | Female (male-sterile) | Yes | α | No | Tucquala Lake, WA | *F. virginiana* ssp. *platypetala* | SRR6149712 |
| GH22 | Male-fertile | No | β | No | Rose Creek, WA | *F. virginiana* ssp. *platypetala* | SRR6149715 |
| KB11 | Male-fertile | No | β | Wei et al. in press | Kamiak Butte, WA | *F. virginiana* ssp. *platypetala* | SRR6149721 |
| NA.CA.12.1.3 | Male-fertile | No | γ | No | Longville, CA | *F. virginiana* ssp. *platypetala* | SRR6149690 |
| NA.CA.12.3.3 | Male-fertile | No | γ | No | Longville, CA | *F. virginiana* ssp. *platypetala* | SRR6149691 |
| NA.OR.3.2.3 | Male-fertile | No | α | No | Walton Lake, OR | *F. virginiana* ssp. *platypetala* | SRR6149724 |
| NA.UT.2.4.4 | Male-fertile | No | γ | No | Lodgepole Campground, UT | *F. virginiana* ssp. *platypetala* | SRR6149726 |
| NA.WA.1.2.3 | Male-fertile | No | α | No | Tucquala Lake, WA | *F. virginiana* ssp. *platypetala* | SRR6149700 |
| NA.WA.1.6.3 | Male-fertile | No | α | No | Tucquala Lake, WA | *F. virginiana* ssp. *platypetala* | SRR6149674 |
| BR105 | Female (male-sterile) | Yes | γ | No | Crawford Co., PA | *F. virginiana* ssp. *virginiana* | SRR6149678 |
| GAM104 | Female (male-sterile) | Yes | α | No | Crawford Co., PA | *F. virginiana* ssp. *virginiana* | SRR6149671 |
| NA.MI.2.12.3 | Female (male-sterile) | Yes | α | No | Wakeley Lake, MI | *F. virginiana* ssp. *virginiana* | SRR6149686 |
| NA.NY.1.13.3 | Female (male-sterile) | Yes | γ | No | Ulster Co., New York | *F. virginiana* ssp. *virginiana* | SRR6149688 |
| NA.NY.1.15.3 | Female (male-sterile) | Yes | α | No | Ulster Co., New York | *F. virginiana* ssp. *virginiana* | SRR6149697 |
| NA.ON.2.17.3 | Female (male-sterile) | Yes | α | No | Wellington County Forest, ON | *F. virginiana* ssp. *virginiana* | SRR6149698 |
| NA.VT.1.1.4 | Female (male-sterile) | Yes | α | No | Bennington Co., VT | *F. virginiana* ssp. *virginiana* | SRR6149670 |
| NA.WI.1.8.3 | Female (male-sterile) | Yes | α | No | Taylor Co., WI | *F. virginiana* ssp. *virginiana* | SRR6149695 |
| Y33b2 | Female (male-sterile) | Yes | α | Spigler et al. 2008 | Crawford Co., PA | *F. virginiana* ssp. *virginiana* | SRR6149709 |
| BR14 | Male-fertile | No | α | No | Crawford Co., PA | *F. virginiana* ssp. *virginiana* | SRR6149677 |
| GAM30 | Male-fertile | No | γ | No | Crawford Co., PA | *F. virginiana* ssp. *virginiana* | SRR6149683 |
| NA.MI.2.14.3 | Male-fertile | No | α | No | Wakeley Lake, MI | *F. virginiana* ssp. *virginiana* | SRR6149687 |
| NA.ON.2.21.3 | Male-fertile | No | α | No | Wellington County Forest, ON | *F. virginiana* ssp. *virginiana* | SRR6149725 |
| NA.PA.1.7.3 | Male-fertile | No | γ | No | Crawford Co., PA | *F. virginiana* ssp. *virginiana* | SRR6149729 |
| NA.VT.1.7.3 | Male-fertile | No | α | No | Bennington Co., VT | *F. virginiana* ssp. *virginiana* | SRR6149711 |
| NA.WI.1.8.4 | Male-fertile | No | α | No | Taylor Co., WI | *F. virginiana* ssp. *virginiana* | SRR6149696 |
| O477 | Male-fertile | No | γ | Spigler et al. 2008 | Crawford Co., PA | *F. virginiana* ssp. *virginiana* | SRR6149693 |
| ^a^For male-fertile plants, clade is defined as the clade of the closest-related female plant, as determined by chloroplast phylogeny, or n/a if undetermined | | | | | | | |
