# Supplementary material for: Repeated translocation of a gene cassette drives sex-chromosome turnover in strawberries
Source: PLoS Biol. 2018 Aug 27;16(8):e2006062. doi: 10.1371/journal.pbio.2006062 (PMC6128632; doi:10.1371/journal.pbio.2006062)
Supplement: S2 Table — (DOCX) [file pbio.2006062.s009.docx]

**S2 Table. Sex-specific 31-mers per group.**

|  | **N Individuals** | **N sex-specific 31-mers** |  |
| --- | --- | --- | --- |
| *F. virginiana* ssp. *virginiana* females | 9 | 77 |  |
| *F. virginiana* ssp. *platypetala* females | 9 | 69 |  |
| *F. chiloensis* females | 9 | 651 |  |
| α clade females | 11 | 71 |  |
| β+γ^a^ clade females | 18 | 77 |  |
| γ clade females | 15 | 284 |  |
| α clade females just *F. virginiana* | 11 | 71 |  |
| β+γ^a^ clade females just *F. virginiana* | 8 | 521 |  |
| γ clade females just *F. virginiana* | 6 | 1259 |  |
| α clade males^b^ | 8 | 1^c^ |  |
| β+γ^a^ clade males^b^ | 20 | 0 |  |
| γ clade males^b^ | 18 | 0 |  |
|  |  |  |  |
| ^a^β and γ clades were combined due to low sample size in β clade | | | |
| ^b^For male-fertile plants, clade is defined as the clade of the closest-related female plant (Fig 3), as determined by chloroplast phylogeny | | | |
| ^c^A near-homopolymer run, CTGCCCCCCTCCCTCCCCCCCCCCCCCCCCC, likely a false positive | | | |
