# Supplementary material for: Repeated translocation of a gene cassette drives sex-chromosome turnover in strawberries
Source: PLoS Biol. 2018 Aug 27;16(8):e2006062. doi: 10.1371/journal.pbio.2006062 (PMC6128632; doi:10.1371/journal.pbio.2006062)
Supplement: S3 Table — (DOCX) [file pbio.2006062.s010.docx]

**S3 Table. Female-specific sequence.**

|  | **Upstream Outer** | **Upstream Flanking** | **SDR cassette** | **Downstream Flanking** | **Downstream Outer** |
| --- | --- | --- | --- | --- | --- |
| α 31-mers^a^ | 0 (0) | 0 (0) | 22 (65) | 0 (0) | 0 (0) |
| β 31-mers^b^ | 0 (0) | 68 (68) | 540 (690) | 363 (501) | 0 (0) |
| γ 31-mers^c^ | 2 (5) | 32 (32) | 70 (70) | 142 (142) | 93 (93) |
| Range^d^ | 0-1836bp | 1837-4808bp | 4809-18574bp | 18575-24627bp | 24628-27928bp |
| Fvb regions^e^ | Fvb6: 37.600-37.602Mb | Fvb6: 13.145-13.149Mb | Fvb6: 1.632-1.642Mb,  Fvb7: 18.504-18.507Mb | Fvb6: 13.128-13.145Mb,  Fvb4: 21.343-21.345Mb | Fvb6: 37.602-37.604Mb |
| Genes^f^ | F−box/kelch | glucan endo−1,3−beta−glucosidase | GDP−mannose 3,5−epimerase 2 (*GMEW*), 60S acidic ribosomal protein P0 (*RPP0W*) | uncharacterized protein,  inactive purple acid phosphotase 16 | arabinogalactan |
|  |  |  |  |  |  |
| ^a^Female-specific 31-mers from the α clade aligning to each section, either as a perfect match or (in parentheses) allowing up to two mismatches | | | | | |
| ^b^Female-specific 31-mers from the β clade aligning to each section, either as a perfect match or (in parentheses) allowing up to two mismatches | | | | | |
| ^c^Female-specific 31-mers from the γ clade aligning to each section, either as a perfect match or (in parentheses) allowing up to two mismatches | | | | | |
| ^d^Definiton of each section relative to the assembled *F.chiloensis* SDR haplotype | | | |  |  |
| ^e^Portion(s) of Fvb reference genome homologous to each section | | | |  |  |
| ^f^Predicted protein products of annotated genes (Fig 4) | | | |  |  |
