# Supplementary material for: Repeated translocation of a gene cassette drives sex-chromosome turnover in strawberries
Source: PLoS Biol. 2018 Aug 27;16(8):e2006062. doi: 10.1371/journal.pbio.2006062 (PMC6128632; doi:10.1371/journal.pbio.2006062)
Supplement: S4 Table — (DOCX) [file pbio.2006062.s011.docx]

**S4 Table. Read pairs aligned to key regions of W haplotype.**

| **Left^a^** | **Right^a^** | **Left Fvb match^b^** | **Right Fvb match^b^** | **α pairs^c^** | **β pairs^c^** | **γ pairs^c^** | **Male pairs^c^** | **Inference of where these sequences are adjacent in genome** |
| --- | --- | --- | --- | --- | --- | --- | --- | --- |
| **Adjacent in SDR, not autosomes; see Fig 4A** | | | | | | | | |
| 1.80 | 1.80 | Fvb6_37.6 | Fvb6_13.1 | 0.0 | 0.0 | 1.1 | 0.0 | only in γ W |
| 4.80 | 4.80 | Fvb6_13.1 | Fvb6_1.6 | 0.0 | 2.0 | 1.3 | 0.0 | only in β and γ W |
| 7.26 | 7.26 | None | Fvb6_1.6 | 0.0 | 3.7 | 0.7 | 0.0 | only in β and γ W |
| 14.38 | 14.38 | Fvb6_1.6 | Fvb7_18.5 | 2.3 | 0.0 | 1.4 | 0.0 | only in α, β (?), and γ W (edge of *RPP0W*) - perhaps absent in β reads by chance |
| 15.59 | 15.59 | Fvb7_18.5 | Fvb6_1.6 | 3.4 | 2.7 | 1.2 | 0.0 | only in α, β ,and γ W (edge of *RPP0W*) |
| 18.57 | 18.57 | Fvb6_1.6 | Fvb6_13.1 | 0.0 | 1.7 | 1.9 | 0.0 | only in β and γ W |
| 22.45 | 22.45 | Fvb4_21.3 | Fvb6_13.1 | 0.0 | 3.3 | 1.4 | 0.0 | only in β and γ W |
| 23.35 | 23.35 | Fvb6_13.1 | Fvb6_13.1 | 0.0 | 2.7 | 1.5 | 0.0 | only in β and γ W; nearly so but too far to span or inverted in autosomal and Z homoeologs to Fvb6_13.1 |
| 24.65 | 24.65 | Fvb6_13.1 | Fvb6_37.6 | 0.0 | 0.0 | 1.3 | 0.0 | only in γ W |
| 25.29 | 25.29 | Fvb6_37.6 | None | 0.0 | 0.0 | 1.9 | 0.0 | only in γ W |
| **Adjacent autosomes, not SDR; see S5 Fig** | | | | | | | | |
| 1.80 | 24.60 | Fvb6_37.6 | Fvb6_37.6 | 20.1 | 12.3 | 10.1 | 11.45 | autosomal and Z homoeologs to Fvb6_37.6, not W SDR haplotype |
| 4.80 | 18.50 | Fvb6_13.1 | Fvb6_13.1 | 19.6 | 23.7 | 10.9 | 11.07 | autosomal and Z homoeologs to Fvb6_13.1, not W SDR haplotype |
| 14.10 | 15.50 | Fvb6_1.6 | Fvb6_1.6 | 13.9 | 10.7 | 10.1 | 8.55 | autosomal and Z homoeologs to Fvb6_1.6, not W SDR haplotype |

^a^position on assembled W haplotype (kb)

^b^best match of this segment to Fvb reference genome

^c^mean number of read pairs per individual spanning these positions
