# Supplementary material for: Repeated translocation of a gene cassette drives sex-chromosome turnover in strawberries
Source: PLoS Biol. 2018 Aug 27;16(8):e2006062. doi: 10.1371/journal.pbio.2006062 (PMC6128632; doi:10.1371/journal.pbio.2006062)
Supplement: S5 Table — (DOCX) [file pbio.2006062.s012.docx]

**S5 Table. Genes within 50kb of the three SDR locations, as per *Fragaria vesca* reference genome *Fvb*.**

| **Gene** | **Fvb Chromosome** | **Start Site** | **End Site** | **Annotation** |
| --- | --- | --- | --- | --- |
| gene16572 | Fvb6 | 1586690 | 1587144 | Chorismate synthase |
| gene16571 | Fvb6 | 1588952 | 1590773 | Auxin-induced protein AUX28 |
| gene16570 | Fvb6 | 1592625 | 1593983 | Sporozoite surface protein 2, Precursor |
| gene16569 | Fvb6 | 1597699 | 1598363 | Auxin-responsive protein |
| gene16568 | Fvb6 | 1599500 | 1599844 | Hormonally up-regulated neu tumor-associated kinase |
| gene16567 | Fvb6 | 1600604 | 1602111 | Disease resistance protein At4g27190 |
| gene16566 | Fvb6 | 1602720 | 1610265 | Putative mediator of RNA polymerase II transcription subunit 14 |
| gene16565 | Fvb6 | 1610884 | 1612896 | Pentatricopeptide repeat-containing protein |
| gene16564 | Fvb6 | 1615046 | 1618371 | Transcription factor UNE12 |
| gene16563 | Fvb6 | 1621307 | 1623847 | Sec-independent protein translocase protein tatA/E |
| gene16562 | Fvb6 | 1624271 | 1625783 | Pentatricopeptide repeat-containing protein |
| gene16561 | Fvb6 | 1626236 | 1629136 | Light-inducible protein CPRF2 |
| gene16560 | Fvb6 | 1630850 | 1632688 | V-type proton ATPase subunit F (V-ATPase subunit F) |
| gene16559 | Fvb6 | 1638510 | 1640658 | GDP-mannose 3,5-epimerase 1 |
| gene16558 | Fvb6 | 1642269 | 1646128 | Alanyl-tRNA synthetase (AlaRS) |
| gene16557 | Fvb6 | 1648738 | 1650141 | Calmodulin-like protein 11 |
| gene16556 | Fvb6 | 1656482 | 1659759 | Probable aldo-keto reductase |
| gene16555 | Fvb6 | 1660190 | 1661982 | Probable aldo-keto reductase |
| gene16554 | Fvb6 | 1663355 | 1666959 | Small nuclear ribonucleoprotein-associated protein B |
| gene16553 | Fvb6 | 1670115 | 1671049 | Pentatricopeptide repeat-containing protein |
| gene16552 | Fvb6 | 1675364 | 1676856 | Probable aldo-keto reductase |
| gene16551 | Fvb6 | 1682365 | 1690973 | Probable aldo-keto reductase |
| gene21942 | Fvb6 | 13082379 | 13082801 | BUD13 homolog |
| gene21943 | Fvb6 | 13085445 | 13088065 | CHLOROPLAST IMPORT APPARATUS 2 |
| gene21944 | Fvb6 | 13094835 | 13095307 | Spermatogenesis-associated protein 18 homolog |
| gene21945 | Fvb6 | 13095846 | 13102693 | Acetylglucosamine--dolichyl-phosphate N-acetylglucosaminephosphotransferase |
| gene21946 | Fvb6 | 13103374 | 13110222 | TAF5-like RNA polymerase II p300/CBP-associated factor-associated |
| gene21947 | Fvb6 | 13115016 | 13116211 | UvrABC system protein C (Protein uvrC) |
| gene21948 | Fvb6 | 13117889 | 13125089 | Endonuclease |
| gene21949 | Fvb6 | 13126882 | 13130834 | Inactive purple acid phosphatase 16 |
| gene21950 | Fvb6 | 13132493 | 13135967 | Biogenesis of lysosome-related organelles complex 1 subunit 2 |
| gene21951 | Fvb6 | 13138013 | 13139492 | Putative defensin-like protein 315 |
| gene21952 | Fvb6 | 13139803 | 13140859 | Hypothetical protein |
| gene21953 | Fvb6 | 13142786 | 13143664 | RNA-directed RNA polymerase |
| gene21954 | Fvb6 | 13145581 | 13146668 | Glucan endo-1,3-beta-glucosidase, Precursor |
| gene21955 | Fvb6 | 13149169 | 13150048 | Glucan endo-1,3-beta-glucosidase, Precursor |
| gene21956 | Fvb6 | 13151083 | 13151913 | Thaumatin-like protein 1 |
| gene21957 | Fvb6 | 13157172 | 13158736 | DNA-directed RNA polymerase II subunit RPB1 |
| gene21958 | Fvb6 | 13160242 | 13160688 | FERM domain-containing protein 7 |
| gene21959 | Fvb6 | 13163221 | 13164403 | Glucan endo-1,3-beta-glucosidase, Precursor |
| gene21960 | Fvb6 | 13165566 | 13170473 | DNA repair protein recA homolog 2, mitochondrial, Precursor |
| gene21961 | Fvb6 | 13176306 | 13179270 | Cytokinin dehydrogenase |
| gene21962 | Fvb6 | 13179777 | 13180133 | Mediator of RNA polymerase II transcription subunit 23 |
| gene21963 | Fvb6 | 13183566 | 13191526 | DnaJ homolog subfamily C member 7 |
| gene21964 | Fvb6 | 13193072 | 13195817 | Nectarin-2, Precursor |
| gene27977 | Fvb6 | 37551773 | 37553910 | Proteasome subunit beta type-7-B, Precursor |
| gene27978 | Fvb6 | 37555127 | 37557605 | Cytochrome P450 724B1 |
| gene27979 | Fvb6 | 37559926 | 37561040 | Methyltransferase-like protein 7B, Precursor |
| gene27980 | Fvb6 | 37561879 | 37563478 | 31 kDa ribonucleoprotein, chloroplastic, Precursor |
| gene27981 | Fvb6 | 37563877 | 37567633 | Transcription factor GTE6 |
| gene27982 | Fvb6 | 37568491 | 37569054 | Knirps-related protein |
| gene27983 | Fvb6 | 37570153 | 37572904 | F-box protein At5g07610 |
| gene27984 | Fvb6 | 37573593 | 37575071 | Putative defensin-like protein 315 |
| gene27985 | Fvb6 | 37579865 | 37581156 | F-box/LRR-repeat protein At2g43260 |
| gene27986 | Fvb6 | 37584293 | 37585472 | F-box protein At5g07610 |
| gene27987 | Fvb6 | 37592499 | 37595391 | Abhydrolase domain-containing protein FAM108C1 |
| gene27988 | Fvb6 | 37596307 | 37598806 | Glycerol-3-phosphate dehydrogenase [NAD+] |
| gene27989 | Fvb6 | 37600362 | 37601141 | Putative F-box protein At5g50220 |
| gene27990 | Fvb6 | 37603601 | 37603957 | Regulatory protein MIG1 |
| gene27991 | Fvb6 | 37604863 | 37607437 | Potassium-transporting ATPase A chain |
| gene27992 | Fvb6 | 37607504 | 37607821 | Branchpoint-bridging protein |
| gene27993 | Fvb6 | 37609995 | 37610339 | Alpha,alpha-trehalose-phosphate synthase [UDP-forming] 1 (AtTPS1) |
| gene27994 | Fvb6 | 37611466 | 37614336 | Putative disease resistance protein RGA4 |
| gene27995 | Fvb6 | 37615513 | 37618075 | Chaperone protein dnaJ |
| gene27996 | Fvb6 | 37620625 | 37620975 | Phosphatidylserine decarboxylase beta chain |
| gene27997 | Fvb6 | 37624162 | 37625046 | Classical arabinogalactan protein 6, Precursor |
| gene27998 | Fvb6 | 37626049 | 37627521 | Pectate lyase, Precursor |
| gene27999 | Fvb6 | 37632278 | 37633769 | Zinc finger protein CONSTANS-LIKE 7 |
| gene28000 | Fvb6 | 37635758 | 37636183 | 17.8 kDa class I heat shock protein |
| gene28001 | Fvb6 | 37637267 | 37637876 | Ubiquitin domain-containing protein DSK2 |
| gene28002 | Fvb6 | 37643515 | 37646267 | Uncharacterized membrane protein At3g27390 |
| gene28003 | Fvb6 | 37647031 | 37647625 | Early nodulin-like protein 1, Precursor |
| gene28004 | Fvb6 | 37650069 | 37655329 | Succinate dehydrogenase [ubiquinone] iron-sulfur subunit 2, mitochondrial (Ip), Precursor |
