# Supplementary material for: Repeated translocation of a gene cassette drives sex-chromosome turnover in strawberries
Source: PLoS Biol. 2018 Aug 27;16(8):e2006062. doi: 10.1371/journal.pbio.2006062 (PMC6128632; doi:10.1371/journal.pbio.2006062)
Supplement: S6 Table — (DOCX) [file pbio.2006062.s013.docx]

**S6 Table. BAC sequencing and assembly data.**

| **BAC** | **ID** | **Index 1** | **Index 2** | **Paired Reads** | **Probes Hit** | **Quality Trim** | **Velvet kmer** | **Contigs >300bp** | **Longest Contig** | **Scaffold Group** | **Homeo-SNP Present in BAC Sequence** | **Linkage Mapped Subgenome** |
| --- | --- | --- | --- | --- | --- | --- | --- | --- | --- | --- | --- | --- |
|  |  |  |  |  |  |  |  |  |  |  |  |  |
| BAC_001 | Bb0011E03 | ATTACTCG | TATAGCCT | 296852 | 1, 2a, 2b, 3 | Q20 | 69 | 1 | 99620 | 1 | Fvb6_1796977 A->G Fvb6_1796987 G->A Fvb6_1797055 C->T | Bi |
| BAC_002 | Bb0012C05 | TCCGGAGA | TATAGCCT | 485396 | 1, 2a, 2b | Q30 | 81 | 1 | 106401 | 2 |  |  |
| BAC_003 | Bb0020D14 | CGCTCATT | TATAGCCT | 348009 | 2a, 2b | Q30 | 49 | 22 | 27537 | 3 |  |  |
| BAC_004 | Bb0021F11 | ATTACTCG | ATAGAGGC | 222731 | 1, 2b, 4 | Q20 | 55 | 22 | 33617 | 3 |  |  |
| BAC_005 | Bb0024C14 | TCCGGAGA | ATAGAGGC | 121098 | 1, 2a, 2b, 4 | Q20 | 85 | 1 | 93178 | 7 |  |  |
| BAC_006 | Bb0024E01 | CGCTCATT | ATAGAGGC | 383761 | 1, 2b, 3 | Q30 | 57 | 3 | 92370 | 4 |  |  |
| BAC_007 | Bb0035H10 | ATTACTCG | CCTATCCT | 313629 | 1, 2a, 2b | Q30 | 37 | 4 | 101321 | 5 | Fvb6_1807915 C->A Fvb6_1794391 C->T | Av |
| BAC_008 | Bb0040P08 | TCCGGAGA | CCTATCCT | 408644 | 1, 2b, 3 | Q30 | 55 | 5 | 89180 | 6 |  |  |
| BAC_009 | Bb0041M08 | CGCTCATT | CCTATCCT | 503475 | 2b, 3 | Q30 | 63 | 2 | 55104 | 7 |  |  |
| BAC_010 | Bb0043P06 | ATTACTCG | GGCTCTGA | 325456 | 1, 2a | Q30 | 43 | 25 | 14804 | 8 | Fvb6_1794282 G->T | B2 |
| BAC_011 | Bb0044F06 | TCCGGAGA | GGCTCTGA | 335947 | 1, 2a, 2b, 3 | Q30 | 63 | 12 | 57086 | 4 |  |  |
| BAC_012 | Bb0044I21 | CGCTCATT | GGCTCTGA | 433561 | 1, 2a, 2b, 3 | Q30 | 47 | 1 | 76795 | 4 |  |  |
| BAC_013 | Bb0058A15 | GAGATTCC | TATAGCCT | 413940 | 1, 2a, 3 | Q30 | 75 | 6 | 32514 | 9 |  |  |
| BAC_014 | Bb0058C13 | ATTCAGAA | TATAGCCT | 230370 | 1, 2a | Q20 | 79 | 1 | 101546 | 7 | Fvb6_1796797 G->A | Bi |
| BAC_015 | Bb0064K21 | GAATTCGT | TATAGCCT | 94669 | 1, 2a | Q30 | 33 | 6 | 73749 | 5 |  |  |
| BAC_016 | Bb0066N24 | GAGATTCC | ATAGAGGC | 73450 | 3,4 | Q30 | 51 | 2 | 82817 | 6 | Fvb6_1626057 A->G | B2 |
| BAC_017 | Bb0067P06 | ATTCAGAA | ATAGAGGC | 135246 | 3 | Q20 | 63 | 26 | 34685 | 10 |  |  |
| BAC_018 | Bb0085J05 | GAATTCGT | ATAGAGGC | 67118 | 2a, 2b | Q20 | 51 | 37 | 15433 | 5 |  |  |
| BAC_019 | Bb0088J18 | GAGATTCC | CCTATCCT | 269738 | 1, 2a, 2b | Q30 | 85 | 71 | 3352 | 3 |  |  |
| BAC_020 | Bb0091H20 | ATTCAGAA | CCTATCCT | 269174 | 1, 3, 4 | Q20 | 59 | 51 | 17777 | 10 |  |  |
| BAC_021 | Bb0091I22 | GAATTCGT | CCTATCCT | 283231 | 1 | Q30 | 69 | 1 | 92940 | 7 | Fvb6_1796797 G->A | Bi |
| BAC_022 | Bb0092K09 | GAGATTCC | GGCTCTGA | 258290 | 1, 2a, 2b, 3 | Q20 | 69 | 40 | 31068 | 7 |  |  |
| BAC_023 | Bb0097B22 | ATTCAGAA | GGCTCTGA | 277625 | 1, 3 | Q20 | 51 | 28 | 44412 | 9 |  |  |
| BAC_024 | Bb0098E20 | GAATTCGT | GGCTCTGA | 282547 | 1, 2a | Q30 | 33 | 3 | 80868 | 8 | Fvb6_1794282 G->T | B2 |
| BAC_025 | Bb0101B08 | CTGAAGCT | TATAGCCT | 142291 | 1, 3 | Q20 | 83 | 9 | 41193 | 9 |  |  |
| BAC_026 | Bb0109C15 | TAATGCGC | TATAGCCT | 104994 | 1, 2a, 2b, 3 | Q20 | 47 | 24 | 34519 | 7 | Fvb6_1796797 G->A | Bi |
| BAC_027 | Bb0116O24 | CGGCTATG | TATAGCCT | 52455 | 4 | Q20 | 33 | 12 | 52931 | 7 |  |  |
| BAC_028 | Bb0119G05 | CTGAAGCT | ATAGAGGC | 116416 | 1, 2a, 4 | Q20 | 71 | 4 | 52780 | 1 | Fvb6_1796977 A->G Fvb6_1796987 G->A Fvb6_1797055 C->T | Bi |
| BAC_029 | Bb0129L08 | TAATGCGC | ATAGAGGC | 91515 | 1, 2a, 2b | Q20 | 59 | 10 | 65381 | 2 |  |  |
| BAC_030 | Bb0142L02 | CGGCTATG | ATAGAGGC | 58724 | 2b | Q20 | 85 | 1 | 97360 | 5 |  |  |
| BAC_031 | Bb0145G13 | CTGAAGCT | CCTATCCT | 153120 | 1, 2a, 2b, 3 | Q20 | 79 | 16 | 68550 | 7 |  |  |
| BAC_032 | Bb0145I02 | TAATGCGC | CCTATCCT | 169219 | 1, 2a, 2b, 3 | Q30 | 45 | 2 | 85828 | 4 |  |  |
| BAC_033 | Bb0149F19 | CGGCTATG | CCTATCCT | 213391 | 1, 2a | Q30 | 67 | 6 | 71946 | 11 | Fvb6_1794416 A->T | B2 |
| BAC_034 | Bb0151G15 | CTGAAGCT | GGCTCTGA | 227693 | no hits | Q30 | 41 | 10 | 44487 | 2 |  |  |
| BAC_035 | Bb0156C11 | TAATGCGC | GGCTCTGA | 1173902 | 2a | Q30 | 83 | 1 | 46583 | unassigned | |  |
| BAC_036 | Bb0158L03 | CGGCTATG | GGCTCTGA | 330163 | 2a, 2b | Q20 | 81 | 9 | 67346 | 3 |  |  |
| BAC_037 | Bb0160A15 | TCCGCGAA | TATAGCCT | 483792 | 1, 2a, 2b | Q30 | 59 | 1 | 53253 | 3 |  |  |
| BAC_038 | Bb0162H22 | TCTCGCGC | TATAGCCT | 483060 | 1, 2b | Q30 | 41 | 11 | 48148 | 11 | Fvb6_1794416 A->T | B2 |
| BAC_039 | Bb0163A10 | AGCGATAG | TATAGCCT | 211716 | 1, 2a, 2b | Q30 | 61 | 1 | 105899 | 5 | Fvb6_1794391 C->T | Av |
| BAC_040 | Bb0164A22 | TCCGCGAA | ATAGAGGC | 288096 | 2b, 3, 4 | Q30 | 73 | 2 | 89997 | 6 | Fvb6_1626057 A->G | B2 |
| BAC_041 | Bb0169H18 | TCTCGCGC | ATAGAGGC | 189356 | 2b, 4 | Q20 | 87 | 1 | 78128 | unassigned | Fvb6_1626137 A->C | Bi |
| BAC_042 | Bb0171N05 | AGCGATAG | ATAGAGGC | 80126 | 1, 2a, 2b, 3 | Q20 | 79 | 3 | 71067 | 4 |  |  |
| BAC_043 | Bb0172M03 | TCCGCGAA | CCTATCCT | 861006 | 3, 4 | Q30 | 63 | 6 | 87576 | 10 |  |  |
| BAC_044 | Bb0173H13 | TCTCGCGC | CCTATCCT | 732676 | 2a, 2b, 3, 4 | Q30 | 75 | 3 | 93868 | 7 | Fvb6_1626065 C->T | Bi |
| BAC_045 | Bb0173N22 | AGCGATAG | CCTATCCT | 542483 | 1, 2a | Q30 | 39 | 3 | 92053 | 11 | Fvb6_1794416 A->T | B2 |
| BAC_046 | Bb0173P01 | TCCGCGAA | GGCTCTGA | 1386091 | 2a, 3 | Q30 | 79 | 9 | 39234 | 9 |  |  |
| BAC_047 | Bb0177I19 | TCTCGCGC | GGCTCTGA | 767807 | 2a, 3, 4 | Q30 | 79 | 6 | 72941 | 10 |  |  |
| BAC_048 | Bb0179N04 | AGCGATAG | GGCTCTGA | 710415 | 1, 2a | Q30 | 55 | 1 | 58504 | 7 | Fvb6_1796797 G->A | Bi |
| BAC_049 | Bb0180F10 | ATTACTCG | AGGCGAAG | 96276 | 2a, 2b, 3 | Q30 | 83 | 1 | 65000 | 4 |  |  |
| BAC_050 | Bb0180N03 | TCCGGAGA | AGGCGAAG | 77757 | 4 | Q30 | 81 | 1 | 37409 | 3 |  |  |
| BAC_051 | Bb0183O24 | CGCTCATT | AGGCGAAG | 91186 | 1, 2a, 2b | Q30 | 51 | 1 | 70318 | 5 | Fvb6_1794391 C->T | Av |
| BAC_052 | Bb0185C15 | ATTACTCG | TAATCTTA | 116224 | 1, 4 | Q30 | 65 | 1 | 102273 | 5 |  |  |
| BAC_053 | Bb0187M20 | TCCGGAGA | TAATCTTA | 364037 | 1, 3 | Q20 | 57 | 1 | 94591 | 1 | Fvb6_1796977 A->G Fvb6_1796987 G->A Fvb6_1797055 C->T | Bi |
| BAC_054 | Bb0187N12 | CGCTCATT | TAATCTTA | 309238 | 1, 3 | Q20 | 89 | 2 | 119444 | 10 |  |  |
| BAC_055 | Bb0189C09 | ATTACTCG | CAGGACGT | 203667 | 1, 2a, 2b | Q20 | 65 | 2 | 122610 | 3 |  |  |
| BAC_056 | Bb0192C19 | TCCGGAGA | CAGGACGT | 434608 | 1, 2a, 2b, 3 | Q20 | 81 | 1 | 112487 | 4 |  |  |
| BAC_057 | Bb0193N17 | CGCTCATT | CAGGACGT | 230476 | 1 | Q20 | 33 | 1 | 70670 | 2 |  |  |
| BAC_058 | Bb0196A10 | ATTACTCG | GTACTGAC | 326747 | 3 | Q20 | 73 | 8 | 72476 | 9 |  |  |
| BAC_059 | Bb0198D09 | TCCGGAGA | GTACTGAC | 619638 | no hits | Q30 | 79 | 1 | 46678 | unassigned | |  |
| BAC_060 | Bb0198L01 | CGCTCATT | GTACTGAC | 647115 | 3 | Q30 | 53 | 6 | 46955 | 10 |  |  |
| BAC_061 | Bb0204M06 | GAGATTCC | AGGCGAAG | 2045 | no hits | not assembled | | |  |  |  |  |
| BAC_062 | Bb0205F06 | ATTCAGAA | AGGCGAAG | 1813 | 1, 2a | not assembled | | |  |  |  |  |
| BAC_063 | Bb0218H07 | GAATTCGT | AGGCGAAG | 1284 | no hits | not assembled | | |  |  |  |  |
| BAC_064 | Bb0224G07 | GAGATTCC | TAATCTTA | 4715 | no hits | not assembled | | |  |  |  |  |
| BAC_065 | Bb0232H20 | ATTCAGAA | TAATCTTA | 2951 | no hits | not assembled | | |  |  |  |  |
| BAC_066 | Bb0234A03 | GAATTCGT | TAATCTTA | 2631 | 4 | not assembled | | |  |  |  |  |
| BAC_067 | Bb0234L08 | GAGATTCC | CAGGACGT | 4936 | no hits | not assembled | | |  |  |  |  |
| BAC_068 | Bb0234N12 | ATTCAGAA | CAGGACGT | 142574 | 2a, 3 | Q20 | 89 | 1 | 93607 | 10 |  |  |
| BAC_069 | Bb0235E21 | GAATTCGT | CAGGACGT | 235953 | 4 | Q30 | 79 | 1 | 119133 | 3 |  |  |
