# Supplementary material for: Repeated translocation of a gene cassette drives sex-chromosome turnover in strawberries
Source: PLoS Biol. 2018 Aug 27;16(8):e2006062. doi: 10.1371/journal.pbio.2006062 (PMC6128632; doi:10.1371/journal.pbio.2006062)
Supplement: S7 Table — (DOCX) [file pbio.2006062.s014.docx]

**S7 Table. Primers for amplicon sequencing.**

| **Targeted Region** | **Forward primer** | **Reverse primer** | **Length** |
| --- | --- | --- | --- |
| BAC8 | TGCTTGGTATATGGGAAAGAGGAAA | ATTATTCGGGAGAGAAATGTTTGCG | 243 |
| BAC8 | CAAACAAATGTAAGGCACGATCTCT | AATAAAATCCGAGAAAAGCCACACC | 428 |
| BAC8 | CGGAGCAATACTTTTCTTCATCAGT | TACACAAATGCAGCCACAATATCAG | 205 |
| BAC8 | ATTGTTTGGAGAAACCAAGACCAG | AGAAAAGGGAGGAAGAAGAGAATGG | 405 |
| BAC8 | AGTGGTATTCCGCCTAGTAATTTGT | AAGGGATCTTTTGAACACGCTTATG | 257 |
| BAC8 | CAAACCAAAAAGAACAGAAGCCAAG | TATGATTTAGCAACCCAACGTCAAG | 531 |
| BAC8 | TAGGTTTATCTCATCTCATGCCCAG | TACCATTACCTCTCTCTCCCTCTTG | 494 |
| BAC8 | ATATTCTCCACACTTCTTCTCCGAC | AACACAGCTACAAAGTACAAACGAG | 505 |
| BAC8 | CTTTATGGAATTGTAGAGTGGCGAG | CTTAGAGCAAAATGAAGAGAGAGGC | 200 |
| BAC8 | TGATAGTCAAAGTCGAGAAGATCCC | AATTAAGCATCAAATGGGTGGGTTG | 202 |
| BAC8 | ATTAACACAACTCATGGGCTACAAC | AGAAAAAGGCACAAGGAGAAAGAAC | 299 |
| BAC8 | TTGATTTGGAAGTCTGGGTTGAAAG | ATGTTTGGTGTTGTTTTGGTGTTTG | 387 |
| BAC8 | AACAGGGATGTTCTTTAACCCTACT | GTACCTCAATCAATCAAAGTGCCAT | 535 |
| BAC6 | CATCCTGTCCCAATGTTTGCTTTTG | TAAATTGATCCTTGTCTGTCTCCCT | 247 |
| BAC6 | CTGCTTCATAGCTTTTACCAAGTCC | ATAATCTCCTCTACAGCCACAGTAA | 428 |
| BAC6 | TTGTTTGAACTCTGAACCATCTCTG | CACTTAGCTTTGATAGTGAACCCAC | 233 |
| BAC6 | TAGCAACAACCTGATTAACGTGGAG | ACAAGTGCTTTTCTATTTAGCGTGT | 350 |
| BAC6 | CAGAGGTTGCATGTCGATTTCTC | TTAGTCGGTTCACTCGGTTGTATAG | 479 |
| BAC6 | GCCTAACTGCCTACAACATTTATCG | GAGAAGAAAGGGGGAAAAGACAAAG | 279 |
| BAC6 | ATTTTCCCAGTTCTGATTCCCATGT | TTCATTCACACTATATCACACCGGC | 325 |
| BAC6 | ATATAGGAAAACGACGCAGACGA | AATTAGGAATAACGCACACACTCAC | 265 |
| BAC6 | TTGTGCATCATCTTCACCATTTCTC | CTCTCAAGGAACCAACATTAGCATC | 505 |
| BAC6 | TTTAGTTAAGCCAAGCGAACATGATA | GTGGAAATGGAATAAAACGAACAGG | 227 |
| BAC6 | TTTCTAGCTCCATCCCTGCTAAATC | CGAGCATTATTCGTTTCCATAACCT | 445 |
| BAC6 | ACTACTTGGACGATGAAAAATGAGC | CGGGTGGATATAAAAACTCTGGAAG | 316 |
| BAC6 | TCATTGTCATGTAACTCCTTCAGGT | ATATCAAGAGACGAAGTGGAAGAGG | 227 |
| BAC6 | TGCTGTTCTTATTTTTGGTTTGCAC | TTGGATGTCTCCGTTATCATGTTTG | 202 |
| BAC6 | GTGTCTTGACCACCATCACTTTAAC | ACTTACTCTCATTCTGGCTTCAACA | 460 |
| Fvb6_715594-716074 | CTAGATATACAAGACTCCCAAGCTCC | AAGTTTGAAGGTCAGATTCCCACAG | 481 |
| Fvb6_1175425-1175739 | ATTTTATGAACGGCTGAAACTCCTC | TCATCATTTGGTTGGAACTTGACTG | 315 |
| Fvb6_1625689-1626053 | GACGGGTTATAGTTTGCTACTTTGG | GTTAATCACATTGCTTCCATGAGCC | 365 |
| Fvb6_1794196-1794661 | GAATTTGATTGCCGAGTACAGAAGG | GGTTGAAGACACACATTTCTCAGTC | 466 |
| Fvb6_2308272-2308803 | AGTAGAACTCGAATCCCTTAGAAGC | TATTTGATGGCTAGGATGGAGACTG | 532 |
| Fvb6_2957021-2957535 | CTACAACAGAAATAGGGGAGAAGGG | TCAAACTGCACTCGTTACTTCATTC | 515 |
| Fvb6_3455573-3456073 | TCTTTTAAGTACCCCCTACCATTGAG | ACTGATCGTCAAAAACATGCAAGAG | 501 |
| Fvb6_4250623-4250900 | CCAGAAAGATATGGGGGATTTTGAC | ATTTTGTAGCATCACTCCCTAGTCC | 278 |
| Fvb6_5118996-5119408 | AATTTCTGTTGCCTGCTACTTTGAG | TCATATAAGGTGTGCCAATGCAATC | 413 |
| Fvb6_6110392-6110882 | TAAGTCGGCTCAGATTACCATTAGG | GAACTGCTTCTACCAACAAGAGATG | 491 |
| Fvb6_7107451-7107924 | CTTGTGTTCAGTGTGCTGTAATCTC | ACTACTGGTTCATCTTCTTCGATCTG | 474 |
| Fvb6_8094143-8094604 | AGAGCTACTGGTATGGTAACTACTG | CTCATAACTTTCTTCCACAAGAGCC | 462 |
| Fvb6_9282787-9283063 | TCAAAGAGGGAAATAAGGAGTGGAG | ACGACAGCTTCTAAAAACAACTGAC | 277 |
| Fvb6_10476878-10477167 | CAGTTTATCAGAGCCATTAGGGAAC | TGCACTACTCCATTCTCAACTTCTC | 290 |
| Fvb6_10737678-10737912 | CCCTTTCTACTGTGGTGAACATTTG | ACTCACTCGCTCCGTAATTCTAATC | 235 |
| Fvb6_11047255-11047622 | CGCATAAAATATCTCTCCTGGTGC | TGATAGACTTGGGACCTTCTTTGAG | 368 |
| Fvb6_11628788-11629249 | CAAATGACCTAAATAGCGGTTCTGG | ATTAACCACCTTTCCTCTCCTCATC | 462 |
| Fvb6_12304280-12304799 | TGAAGAGAGTAGCAGTAGTTTCAGG | AACCAAAATTCCTCATCTCACACAC | 520 |
| Fvb6_13184204-13184715 | GGTTCAAGTTCATGTTTTCATTCGC | CTTCCATCTTTACCTTTCACTCCAC | 512 |
| Fvb6_14561011-14561468 | GAGTGATAAACCCATCTCATTCAGC | AATACGAAACCACATAAGCAAAGGC | 458 |
| Fvb6_15652157-15652668 | CACATCCTTGCTTGTACTCATCTTC | CTATTACATCTATTGCAGAACCGCC | 512 |
| Fvb6_17605268-17605706 | TCTCTCTCTAACTCGTGCCTTTTAC | ACTTTGGAGCTTTTACCATTTCCAG | 439 |
